# RPA70 depletion induces hSSB1/2-INTS3 complex to initiate ATR signaling

**DOI:** 10.1093/nar/gkv369

**Published:** 2015-04-27

**Authors:** Ananya Kar, Manpreet Kaur, Tanushree Ghosh, Md. Muntaz Khan, Aparna Sharma, Ritu Shekhar, Akhil Varshney, Sandeep Saxena

**Affiliations:** National Institute of Immunology, Aruna Asaf Ali Marg, New Delhi-110067, India

## Abstract

The primary eukaryotic single-stranded DNA-binding protein, Replication protein A (RPA), binds to single-stranded DNA at the sites of DNA damage and recruits the apical checkpoint kinase, ATR via its partner protein, ATRIP. It has been demonstrated that absence of RPA incapacitates the ATR-mediated checkpoint response. We report that in the absence of RPA, human single-stranded DNA-binding protein 1 (hSSB1) and its partner protein INTS3 form sub-nuclear foci, associate with the ATR-ATRIP complex and recruit it to the sites of genomic stress. The ATRIP foci formed after RPA depletion are abrogated in the absence of INTS3, establishing that hSSB-INTS3 complex recruits the ATR-ATRIP checkpoint complex to the sites of genomic stress. Depletion of homologs hSSB1/2 and INTS3 in RPA-deficient cells attenuates Chk1 phosphorylation, indicating that the cells are debilitated in responding to stress. We have identified that TopBP1 and the Rad9-Rad1-Hus1 complex are essential for the alternate mode of ATR activation. In summation, we report that the single-stranded DNA-binding protein complex, hSSB1/2-INTS3 can recruit the checkpoint complex to initiate ATR signaling.

## INTRODUCTION

Exposure to genomic insults causes the activation of apical checkpoint kinases, Ataxia telangiectasia mutated (ATM) and Ataxia telangiectasia and Rad3-related protein (ATR). While ionizing gamma radiation, which causes DNA double-strand breaks (DSBs), activates ATM, UV radiation and replication stress lead to generation of stretches of single-stranded DNA (ssDNA) causing ATR activation. The role of checkpoint kinase, Chk1, as a key signal transducer was soon realized and significant efforts were made to identify the kinase responsible for Chk1 activation (1,2). It was observed that hydroxyurea (HU)-induced phosphorylation of Chk1 was abrogated in cells treated with caffeine but not in immortalized fibroblasts lacking ATM (3). It was also demonstrated that Chk1 is phosphorylated by ATR *in vitro* and UV-induced phosphorylation of Chk1 is reduced in cells expressing kinase-inactive ATR. In response to genotoxic agents, Chk1 was phosphorylated on Serine 317 and 345 in an ATR-dependent manner and mutations at these residues resulted in poor Chk1 activation (4). Thus, these observations establish that exposure to genotoxic agents results in ATR-mediated phosphorylation of Chk1.

ATR activation leading to Chk1 phosphorylation occurs in response to diverse forms of DNA damage. UV-irradiation leads to accumulation of cyclobutane pyrimidine dimers (CPD) and 6–4 photoproducts (6–4PP) that are removed by the nucleotide excision repair machinery and the recruitment of RPA to the undamaged single-stranded DNA results in ATR activation (5). On the other hand, gamma radiation-induced DNA DSBs undergo resection during DNA repair and the subsequently generated single-stranded DNA are coated by RPA, which then recruits ATR to initiate checkpoint signaling (6). Replication stress, broadly defined as slowing or stalling of replication fork progression, is caused by the uncoupling of replicative helicase and DNA polymerases, resulting in stretches of single-stranded DNA (ssDNA) bound by RPA (7). The depletion of nucleotides and replication factors also stalls the replication fork, activating the replication stress response (8). The existence of ssDNA bound RPA next to newly replicated DNA serves as a signal for the recruitment of ATR and checkpoint activation. Therefore, a checkpoint response similar to the one induced after DNA damage is also initiated on replication fork stalling, resulting in Chk1 phosphorylation without actual DNA strand breakage. However, if the replication stress persists, the attempts to stabilize and restart the stalled fork may fail, resulting in fork collapse and DSBs, which would also trigger the ATR activation. Therefore, Chk1 activation usually, but not always, reflects DNA damage.

Single-stranded DNA (ssDNA) is a crucial intermediate generated during several physiological processes such as DNA replication, transcription and recombination. Human genome encodes multiple ssDNA-binding proteins (SSBs) that carry out the essential function of stabilizing the ssDNA: the primary SSB in eukaryotes, replication protein A (RPA), is a heterotrimer comprising of RPA70, RPA32 and RPA14 subunits, and is widely believed to mediate both DNA replication and DNA repair pathways (9,10). It is believed that ATR activation pathway initiates with the binding of RPA to the ssDNA generated at the sites of DNA damage. RPA coated ssDNA then recruits ATR via its partner protein called ATR-interacting protein (ATRIP) (11,12). Simultaneously, the checkpoint clamp loader Rad17-RFC complex loads Rad9-Hus1-Rad1 checkpoint clamp (9–1–1) to the ssDNA, followed by binding of topoisomerase binding protein 1 (TopBP1) (13). Neighboring RPA complexes bind to the checkpoint protein recruitment (CRD) domains of ATRIP and Rad9 bringing TopBP1 in close proximity to activate ATR (14,15).

It has been reported that depletion of RPA results in the loss of checkpoint response and therefore it is widely accepted that RPA is essential for recruiting the ATR-ATRIP complex to the sites of DNA damage (11). However, it has also been reported that ATRIP mutants that have lost the ability to interact with RPA are competent in initiating a checkpoint response (14–18). It was also demonstrated that RPA70 depletion did not prevent the hydroxyurea- or UV-induced phosphorylation of Chk1, though the authors could not rule out the possibility that a low threshold level of RPA was sufficient to activate ATR in their experiments (19). Moreover, the regulation of ATR activity by factors such as CDC6, ATM and MRN complex suggests that there could be independent ways of ATR activation (20–22). One of the possibilities that have not been addressed is whether other SSBs can recruit the checkpoint proteins to the sites of damage. Human genome also encodes other SSBs: single-stranded DNA-binding protein 1/ 2 (hSSB1, hSSB2) and a mitochondrial SSB that have a single oligonucleotide/oligosaccharide-binding fold and resemble the bacterial and archaeal SSB group (10,23). It was observed that in the absence of hSSB1 or hSSB2, activation of ATM in response to ionizing gamma radiation was abrogated (23,24). It was demonstrated that both hSSB1 and hSSB2, independently form a heterotrimeric complex with two other proteins, INTS3 and C9ORF80, where INTS3 serves as a central adaptor required for complex assembly and recruitment to DNA ends (25–27). Though there is disagreement at the stage where hSSB1 acts, it is well accepted that it is essential for the activation of checkpoint proteins like ATM and recruitment of repair proteins like Rad51 after gamma radiation-induced DSBs (25–29). Absence of hSSB1 results in indirect loss of ATR signaling due to the attenuation of DSB response but it is not known if hSSB1 can recruit the ATR-ATRIP complex to ssDNA (30). In the present study, we utilized RNA interference (RNAi) to decrease the endogenous RPA70 and investigated the role of other SSBs in mediating ATR signaling. We report that replication stress resulting from acute depletion of RPA70 induces hSSB1/2-INTS3-mediated ATR activation.

## MATERIALS AND METHODS

### Cell culture, irradiation, cell cycle analysis, drug treatment and cloning

Cell lines were maintained at 37°C in Dulbecco's modified Eagle's medium (DMEM) supplemented with 10% fetal bovine serum and 1% antibiotic solution. For UV-irradiation, medium was removed, the cells were washed with 1x phosphate buffered saline (PBS) followed by exposure to 25 J/m^2^ UV radiation in uncovered dishes with UV-C using UV cross-linker CL-1000 from UVP. Fresh medium was added back to the dishes and the cells were harvested 2 h later. For ionizing radiation treatment, cells were exposed to 10 Gy of gamma radiation and were harvested 2 h later. For cell cycle analysis, the cells were fixed with 70% ethanol after washing with 1x PBS. After fixation, the cell pellet was resuspended in 1x PBS with 0.1% Triton X-100, 20 mg/ml RNase A and 70 mg/ml propidium iodide and then the stained cells were analyzed by flow cytometry. The flow cytometry data were acquired on Becton Dickinson FACSCalibur machine by Cell Quest Pro software. The cell cycle analysis was done by Dean-Jett-Fox method using the FlowJo software. Quantity One Software (version 4.6.3; Bio-Rad) was utilized to evaluate the levels of specific proteins, which were expressed after normalization with the protein loading control. The results were presented as mean ± standard error of the mean (SEM) and Student's *t*-test was used for statistical analysis. *P-*value of less than 0.05 was considered significant, unless noted otherwise. For HU treatment, cells were grown in DMEM supplemented with 0.2 mM HU for 4 h after which the cells were harvested for immunoblotting. For cisplatin treatment, cells were grown in DMEM containing 0.2 μM cisplatin and were harvested after 8 h for immunoblotting. The ATRIP interacting domain of ATR (1–400 aa) and RPA70 were cloned in HA-tagged pCDNA3 vector while hSSB1 was cloned in Myc-tagged pCDNA3 vector. Myc-INTS3 and HA-ATRIP were generous gifts from Junjie Chen and David Cortez respectively.

### Immunoblotting, immunoprecipitation and indirect immunofluorescence

Cells of approximately equal confluency were lysed in proportionate volume of Laemmli buffer for immunoblotting. For immunoprecipitation studies 293T cells were lysed in ice cold NETN buffer (150 mM NaCl, 2 mM EDTA, 50 mM Tris-Cl, 0.2% NP-40 supplemented with protease inhibitors). The cell lysates were first incubated with the specific antibody overnight followed by binding to protein A sepharose for 2 h and after washing, the bound proteins were eluted in Laemmli buffer. For indirect immunofluorescence, HeLa cells grown on coverslips were fixed with 4% formaldehyde for 10 min and permeabilized with 0.2% Triton X-100. Samples were blocked with 10% fetal bovine serum for 30 min and then stained with specific primary antibody for 1 h, followed by incubation with secondary antibody for 1 h. Finally, the cells were visualized under the microscope after mounting with Vectashield mounting reagent containing 4′, 6-diamidino-2-phenylindole (DAPI) that stains the nucleus. The secondary antibodies used were conjugated to Alexa Fluor 488, Alexa Fluor 555 or Alexa Fluor 594 purchased from Invitrogen. To visualize hSSB1, INTS3 and ATRIP foci, cells grown on the coverslips were fixed with cold methanol on ice for 10 min instead of 4% formaldehyde. Immunofluorescence images were captured using the Zeiss LSM 510 confocal microscope. For BrdU immunofluorescence, asynchronously growing HeLa cells were pulsed with 100 μM BrdU for 30 min, washed and exposed to 25 J/m^2^ UV followed by fixation with 4% formaldehyde 2 h later. The fixed cells were treated with 2 M HCl followed by neutralization with 0.1 M sodium borate buffer (pH 8.5). The fixed cells were permeabilized with 0.2% Triton X-100, blocked with 3% BSA and incubated with mouse anti-BrdU antibody.

### Antibodies

Antibodies against RPA70 (Ab1, Ab2), TopBP1, BrdU, DNA Polymerase alpha (αp180) and P-Chk1 Ser317 (Ab2) were purchased from Abcam. Anti-hSSB1, INTS3 and ATRIP antibodies were obtained from Bethyl laboratories. Anti-β-actin, Chk1 and GST antibodies were procured from Santa Cruz Biotechnology Inc. Anti-ATR, P-Chk1 Ser345 (Ab1, Ab2), P-Chk1 Ser317 (Ab1), Chk1 and MRE11 antibodies were purchased from Cell Signaling Technology. Antibodies used to detect the Myc and HA epitopes were obtained from Sigma. Additional antibodies used for immunofluorescence include: goat polyclonal anti-INTS3 (Santa Cruz Biotechnology Inc.); mouse monoclonal anti-RPA70 (Abcam) and rabbit polyclonal anti-HA (Novus Biologicals). Anti-hSSB2 was a generous gift from Dr. Kum Kum Khanna.

### RNAi silencing, cell synchronization and reverse transcription PCR

Transfection of small interfering RNA (siRNA) targeting the endogenous genes was carried out using Lipofectamine 2000 (Invitrogen). 40–80 nM of specific siRNA duplexes were transfected on three consecutive days and the cells were harvested 24 h after the last transfection for immunoblotting or reverse-transcriptase polymerase chain reaction (PCR). To study the S-phase dependence for checkpoint activation, HeLa cells were incubated with 10 μM mevastatin for 18 h to block the cells in G1-phase. Synchronized cells were then transfected with siRNA duplexes targeting *RPA70* or *GL2* on three consecutive days and then harvested. One set of cells was washed with 1x PBS, released into drug free medium and harvested 8 h or 12 h later for western blotting and cell cycle analysis. For reverse-transcriptase PCR, RNA was isolated using the standard TRIzol method and 1 μg of RNA was used for cDNA synthesis. The sequences targeting the human genes were as follows: GL2:CGTACGCGGAATACTTCGA; RPA70(1):GGAATTATGTCGTAAGTCA; RPA70(2):CTGGTTGACGAAAGTGGTG; HSSB1:CCAACAAGGCGGTGCAGAA; HSSB1(2):CCAACAAGGCGGTGCAGAA; HSSB2:CGTGCAAAGTAGCAGATAA; INTS3:GGACAAAGTACTCCAGCTA; INTS3(2):CCAAGCGAGCTGTGACGAA; MRE11:GCTAATGACTCTGATGATA; NBS1:GAAGAAACGTGAACTCAAG; ATRIP:GGTCCACAGATTATTAGAT; ATRIP(2):GGTCCACAGATTATTAGAT; CHK1:CAAGATGTGTGGTACTTTA, GAGAAGGCAATATCCAATA, CCACATGTCCTGATCATAT & GAAGTTGGGCTATCAATGG; ATR:CCTCCGTGATGTTGCTTGA; ATR(2):CCTCCGTGATGTTGCTTGA; TOPBP1:CTCACCTTATTGCAGGAGA; TOPBP1(2):ACGAGTATACAGAGACCTT; RAD9:GTCTTTCCTGTCTGTCTTC; RAD17:CAGACTGGGTTGACCCATC; RAD17(2):CAGACTGGGTTGACCCATC; CLASPIN:ACCTTGCTTAGAGCTGAGT. DNA Polymerase alpha (αp180):CAGGTCGAGAGTACAGAAG. Detailed protocol and the DNA primers used for our experiments are available on request.

### In vitro single-strand binding assay

20 picomoles of biotin labeled or non-biotin labeled 80 base pair single-stranded oligonucleotide (5′TTTTTTTTTTTTTTTTTTTTTTTTTTTTTTCTCCCTTCTTCTCCTCCCTCTCCCTTCCCTTTTTTTTTTTTTTTTTTTTT3′) was incubated with streptavidin-agarose beads for 30 min at 30°C in binding buffer (10 mM Tris, pH 7.5, 150 mM NaCl, 2 mM MnCl_2_, 10 mM MgCl_2_, 0.2 mM EDTA, 10% glycerol, 0.1% NP-40, 10 μg/ml bovine serum albumin and 10 mM DTT). Myc-tagged hSSB1 & INTS3 and HA-tagged ATRIP expressed in 293T cells were purified by immunoprecipitation using anti-Myc and anti-HA antibodies on protein A sepharose beads followed by elution with Myc and HA peptides respectively. Myc-hSSB1 and Myc-INTS3 were pre-incubated in binding buffer at room temperature for 45 min prior to incubation with streptavidin-agarose beads coated with ssDNA for 20 min at 30°C.The hSSB1-INTS3 coated beads were then incubated with purified HA-tagged ATRIP for 30 min at 30°C. The beads were then washed thrice before eluting the bound proteins in Laemmli buffer.

## RESULTS

### RPA depletion-induced genomic stress causes the single-strand binding protein complex, hSSB-INTS3, to form punctate foci

**Figure 1.**
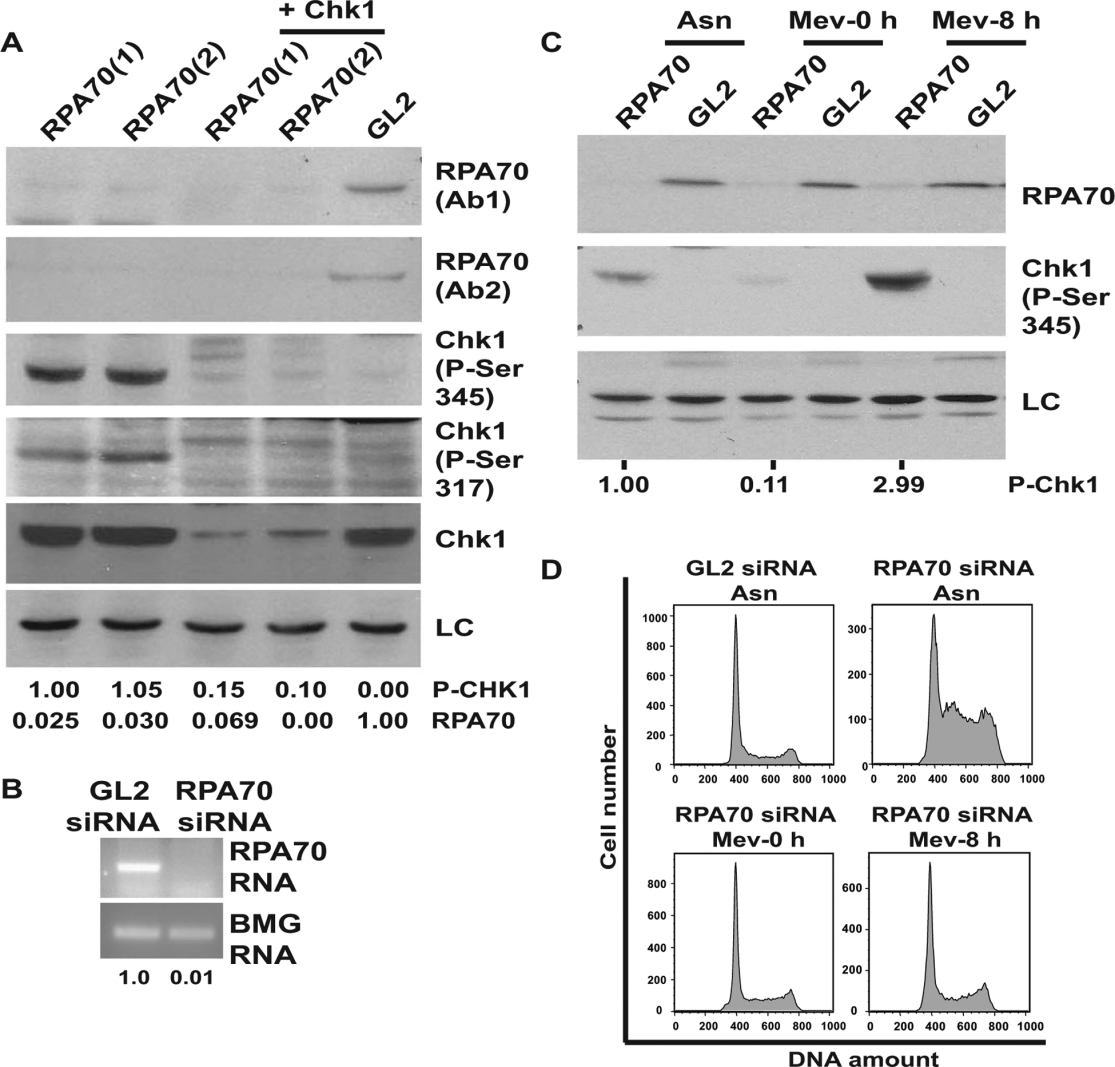
RPA70 depletion-induced phosphorylation of Chk1. (**A**) HeLa cells were transfected on three consecutive days with control *GL2* or siRNA targeting two different regions of RPA70 (*RPA70(1), RPA70(2)*) in combination with *CHK1* siRNA as indicated. Level of RPA70 was evaluated by immunoblotting with two different antibodies (Ab1 and Ab2). Phosphorylation of Chk1 at Ser345 and Ser317 was assessed by specific antibodies. LC refers to the loading control, a non-specific band that displays equal protein load in different lanes. The numbers indicate levels of phosphorylated-Chk1 (P-Chk1) relative to *RPA70(1)* siRNA transfected cells and normalized with the protein loading control. (**B**) HeLa cells were transfected with control *GL2* or *RPA70* siRNA and the level of *RPA70* mRNA was quantified. The numbers indicate the *RPA70* mRNA levels following *RPA70* siRNA depletion relative to control *GL2* transfected cells. β-2 microglobulin (BMG) serves as the internal RNA loading control. (**C**) RPA70 deficiency-induced Chk1 activation is dependent on active DNA replication. *GL2* or *RPA70* siRNA was transfected either in asynchronous cells (Asn) or cells blocked in G1-phase with mevastatin treatment. After transfection on two consecutive days, the cells were either harvested in the presence of mevastatin (Mev-0 h) or 8 h after removal of mevastatin (Mev-8 h) and the levels of RPA70 and phosphorylated-Chk1 were assayed. The numbers indicate phosphorylated-Chk1 levels after different treatments relative to asynchronous *RPA70* siRNA transfected cells. (**D**) Flow cytometry of propidium iodide stained DNA from HeLa cells demonstrates that RPA70 depletion induces S-phase accumulation, which is prevented in the presence of mevastatin.

**Figure 2.**
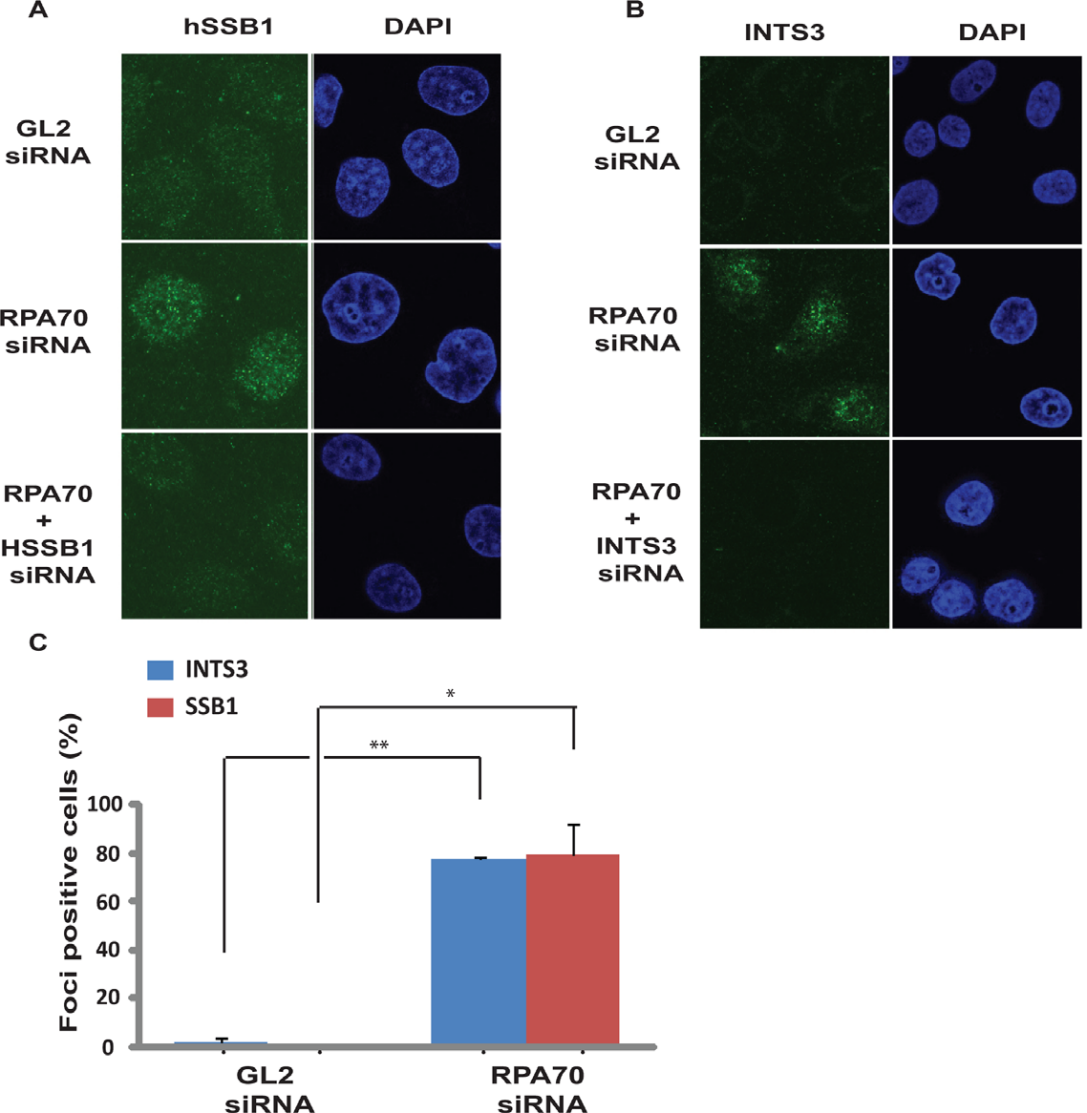
INTS3 and hSSB1 form sub-nuclear foci after RP70 depletion. (**A** and **B**) HeLa cells transfected on three consecutive days with *GL2* or *RPA70* siRNA in combination with *HSSB1* or *INTS3* siRNA as indicated were visualized for hSSB1 and INTS3 foci by immunofluorescence with rabbit anti-hSSB1 and goat anti-INTS3 antibodies respectively. Right panel displays the DAPI staining for each sample. Co-depletion of hSSB1 (A) or INTS3 (B) along with RPA70 confirms that the immunofluorescence signal is from the respective proteins. (**C**) Quantification of INTS3 and hSSB1 foci observed in the experiments described in parts A and B. Cells from *GL2* or *RPA70* siRNA transfected samples were scored for INTS3 and hSSB1 foci and are expressed as a percentage of total cells from each group. Data are represented as the mean ± SE. *P-*values were calculated using two-tailed *t*-test which displays that *RPA70* siRNA transfected samples are significantly different from control *GL2* siRNA transfected samples (*P-value < 0.05; **P-value < 0.005).

Depletion of RPA70 in asynchronous cells resulted in phosphorylation of Chk1 at Ser345 as well as Ser317, both of which are known to be mediated by ATR (Figure 1A and B) (3). Different siRNA duplexes against RPA70 (*(RPA70(1)* and *RPA70(2)*) led to phosphorylation of Chk1 at Ser345 and Ser317, ruling out off-target effects and co-depletion of Chk1 authenticated the phosphorylated-Chk1 band on immunoblots. Moreover, co-expression of RNAi resistant RPA70 suppressed the phosphorylation of Chk1 (Supplementary Figure S1C). Apart from HeLa cells, RPA70 depletion in cell lines of different lineages resulted in Chk1 phosphorylation, which was also confirmed using multiple antibodies (Supplementary Figure S1A and B). RPA70 depletion leads to S-phase accumulation and in order to verify whether RPA70 deficiency-induced Chk1 phosphorylation occurs during the S-phase, we utilized mevastatin to block the cells in G1-phase and then carried out *RPA70* siRNA transfection (Figure 1C) (31). We observed that Chk1 phosphorylation was significantly decreased in G1 blocked RPA70-depleted cells (Figure 1D and Supplementary Figure S5). It is known that cells released from G1 block caused by statins progress slowly into the cell cycle and we observed that as a subpopulation of RPA70-depleted cells entered S-phase 8 h after the removal of mevastatin, Chk1 was phosphorylated (32). Therefore, the RPA depletion-induced Chk1 phosphorylation occurs during DNA replication, presumably because of replication fork stalling (Supplementary Figure S1D) (15,32). It has been reported that depletion of a second human SSB, hSSB1, abrogates the DSB response, so we assayed if it mediates ATR activation (23). We observed that hSSB1 formed punctate foci in RPA70-depleted cells similar to what has been reported after gamma-irradiation, indicating the localization of hSSB1 at the sites of genomic stress (Figure 2A and C and Supplementary Figure S2A). The foci were absent after co-depletion of hSSB1 along with RPA70, confirming that the observed immunofluorescence signal was from hSSB1. HSSB1 is part of the single-stranded DNA-binding complex, wherein its partner protein, INTS3 serves as a central adaptor required for assembly of the complex and recruitment of hSSB1 to the sites of DNA damage (26,27). We observed that after RPA70 depletion INTS3 also formed punctate foci, similar to hSSB1 (Figure 2B and C and Supplementary Figure S2A and B). Therefore, we demonstrate that alternate single-strand binding protein complex, hSSB1-INTS3, forms punctate foci after RPA depletion.

### Chk1 phosphorylation after RPA70 depletion-induced genomic stress is dependent on hSSB1/2-INTS3

**Figure 3.**
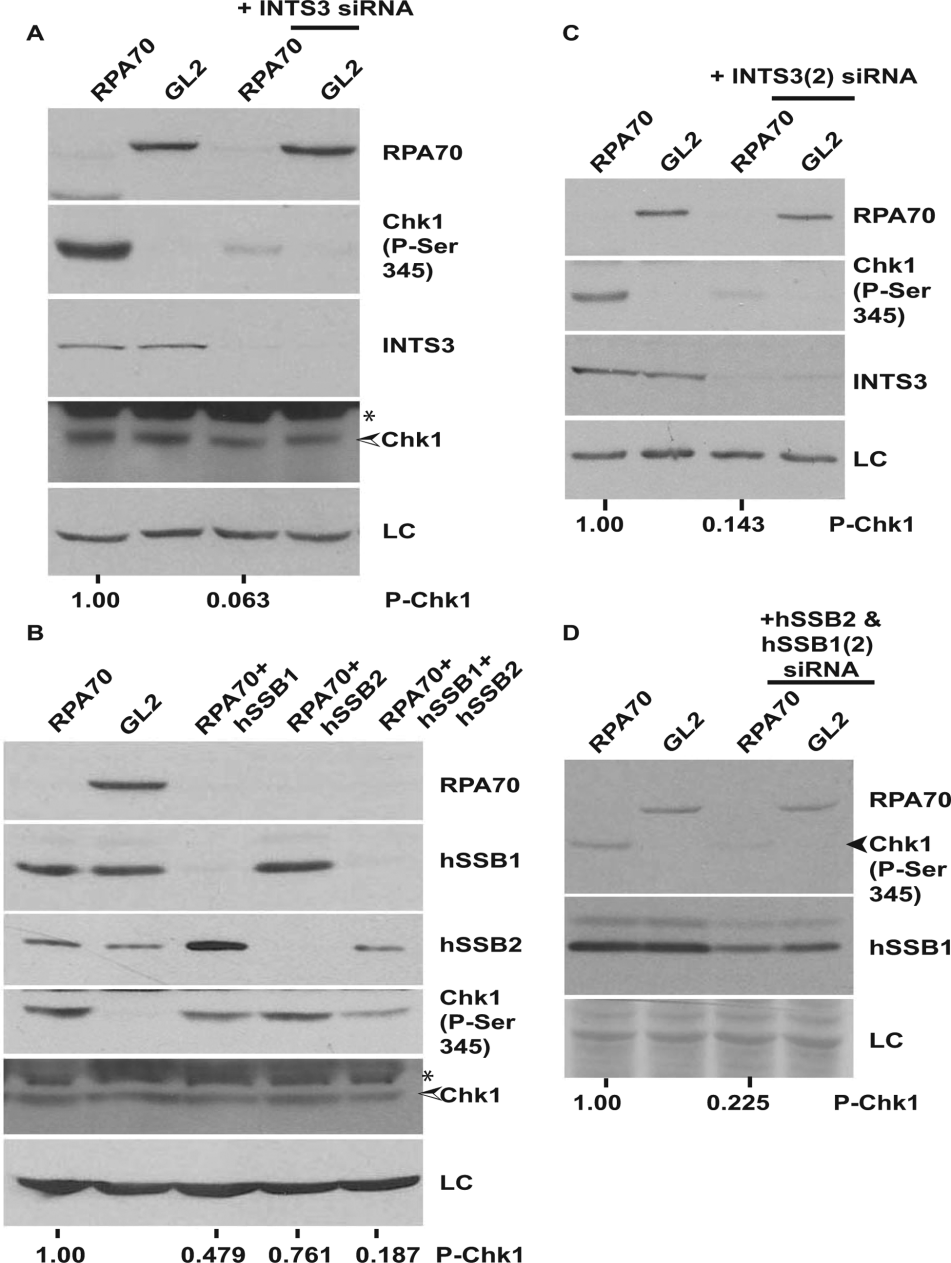
Chk1 phosphorylation in the absence of RPA70 is dependent on single-strand binding protein complex, hSSB1/2-INTS3. (**A**) HeLa cells were transfected on three consecutive days with *GL2* or *RPA70* siRNA in combination with *INTS3* siRNA as indicated and the levels of RPA70, INTS3, total and phosphorylated-Chk1 were assayed. (**B**) HeLa cells were transfected on three consecutive days with *GL2* or *RPA70* siRNA in combination with *HSSB1* and/or *HSSB2* siRNAs as indicated and the levels of RPA70, hSSB1, hSSB2, total and phosphorylated-Chk1 were assayed. * points to a cross-reactive band while LC refers to the protein loading control. The numbers in parts A and B indicate phosphorylated-Chk1 levels following RPA70 depletion alone or in combination with INTS3 or hSSB1 & 2 after normalization with the protein loading control. (**C** and **D**) Different siRNA duplexes confirm the requirement of the hSSB1/2-INTS3 complex for Chk1 phosphorylation in RPA-depleted cells. HeLa cells were transfected on three consecutive days with *GL2* or *RPA70*siRNA in combination with different siRNA duplexes (*INTS3(2), HSSB2* & *HSSB1(2)*) than used in parts A and B. The numbers in parts C and D indicate phosphorylated-Chk1 levels following RPA70 depletion alone or in combination with INTS3 or hSSB1 & 2 after normalization with the protein loading control.

**Figure 4.**
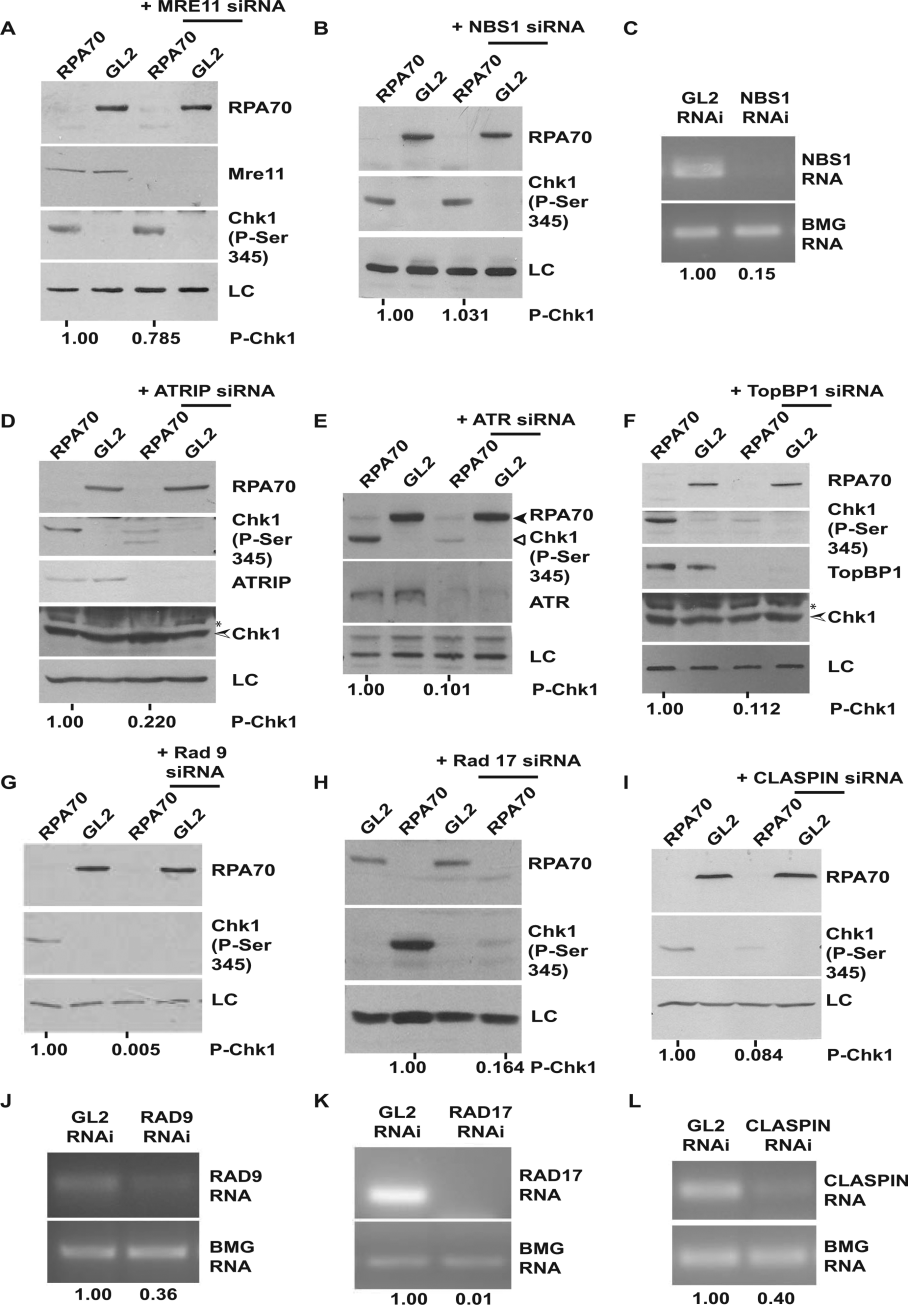
Chk1 phosphorylation in absence of RPA70 is mediated by the ATR-ATRIP complex. HeLa cells were transfected on three consecutive days with *GL2* or *RPA70* siRNA in combination with *MRE11, NBS1, ATRIP, ATR, TOPBP1, RAD9, RAD17* or *CLASPIN* siRNA as indicated and the levels of RPA70, total and phosphorylated-Chk1 were assayed. * points to a cross-reactive band while LC refers to the protein loading control. The numbers in parts A, B, D, E, F, G, H and I indicate phosphorylated-Chk1 levels following RPA70 depletion alone or in combination with other proteins after normalization with the protein loading control. The decrease of MRE11, ATRIP, ATR and TOPBP1 proteins were confirmed by immunoblotting. The decrease of *NBS1* (C), *RAD9* (J), *RAD17* (K) and *CLASPIN* (L) mRNAs were confirmed by reverse-transcriptase PCR and the numbers indicate the mRNA levels following specific siRNA depletion relative to control *GL2* transfected cells. β-2 microglobulin (BMG) serves as the internal RNA loading control.

In order to assess the requirement of hSSB1/2-INTS3 complex in Chk1 phosphorylation, hSSB1 or INTS3 were silenced along with RPA. We observed that co-depletion of INTS3 suppressed the RPA70 depletion-induced Chk1 phosphorylation (Figure 3A and C). As reported previously, we observed that INTS3 depletion inhibited gamma radiation-induced checkpoint response (Supplementary Figure S2B) (27). We also observed that depletion of hSSB1 led to a compensatory increase in the hSSB2 protein level (Figure 3B) (33). Co-transfection of hSSB1 and hSSB2 siRNAs decreased hSSB1 levels but similar hSSB2 levels were observed in comparison to control cells, causing partial suppression of RPA70 RNAi-induced Chk1 phosphorylation (Figure 3B and D). These results indicate that the presence of either hSSB1 or hSSB2 is sufficient for mediating the phosphorylation of Chk1. A counter explanation for our results could be that the suppression of Chk1 phosphorylation after INTS3 depletion is due to disruption of the DSB response but we observed that key DSB response proteins were dispensable for RPA70 depletion induced Chk1 phosphorylation (Figure 4A–C).

It has been previously reported that RPA70 binds independently to ATRIP and Rad9, bringing them in close vicinity and finally leading to activation of ATR by TopBP1. To test if RPA70-independent Chk1 phosphorylation utilizes these factors, a systematic depletion of known checkpoint proteins was carried out. Co-depletion of ATRIP, ATR, TopBP1 or Rad17–9–1–1 complex suppressed the RPA70 depletion-induced Chk1 phosphorylation (Figure 4D–L and Supplementary Figure S3A–E). RNAi-mediated silencing of claspin, which is known to be essential for ATR-dependent Chk1 phosphorylation, also resulted in suppression of Chk1 phosphorylation after RPA depletion (Figure 4I) (34). Therefore, apart from RPA, this novel mode of ATR activation is mediated by the same factors that are involved in the canonical pathway of ATR activation.

### ATR-ATRIP complex associates with the single-strand binding protein complex, hSSB1/2-INTS3

**Figure 5.**
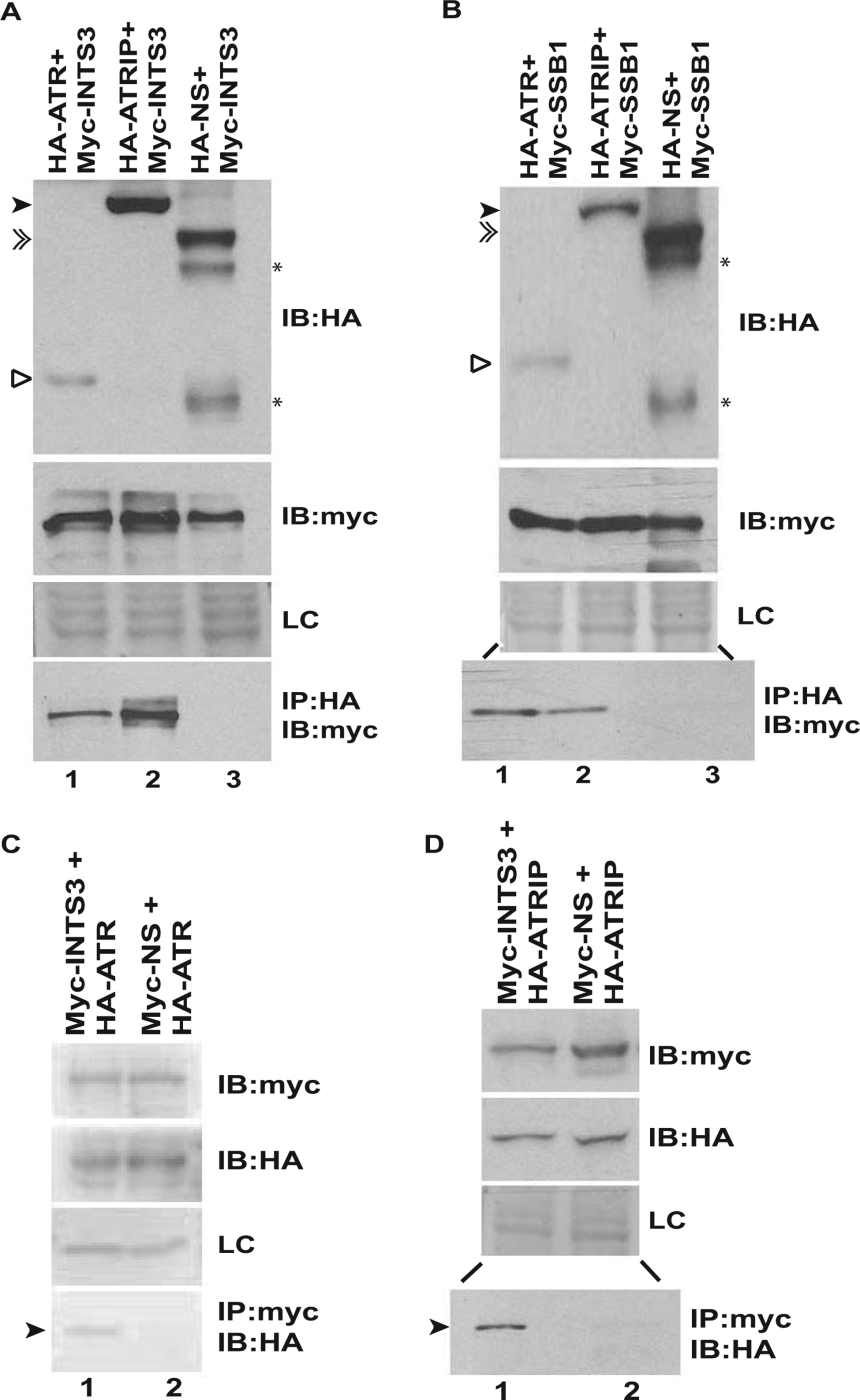
ATR-ATRIP complex associates with hSSB1/2-INTS3. (**A** and **B**) 293T cells were co-transfected with plasmids expressing HA-ATR (1–400 aa), HA-ATRIP or a non-specific protein (HA-NS) in combination with Myc-INTS3 or Myc-hSSB1 as indicated followed by immunoprecipitation (IP) with HA antibody. The lysates were normalized for the expression of HA-tagged and Myc-tagged proteins, shown by immunoblotting (IB) in the first and second panels, respectively. The bottom panel displays the co-immunoprecipitated Myc-tagged protein. HA-ATR (hollow arrowhead), HA-ATRIP (black arrowhead) and HA-NS (double arrowhead) have been marked while (*) displays multiple expression products of the non-specific protein (HA-NS). The expression of an endogenous non-specific protein (LC) in different transfected samples as visualized by Ponceau S staining has been shown. Note that lanes 2 and 3 of the bottom panel in part B are separated by an intervening lane to prevent any spill over artifacts. (**C** and **D**) 293T cells were co-transfected with plasmids expressing HA-ATR (1–400 aa) or HA-ATRIP in combination with Myc-INTS3 or a non-specific protein (Myc-NS) as indicated followed by immunoprecipitation with Myc antibody. The expression of Myc-tagged and HA-tagged proteins have been shown in the first and second panels respectively while the bottom panel displays the co-immunoprecipitated HA-tagged protein (black arrowhead). Myc-INTS3 and Myc-NS have similar mobility. Note that lanes 1 and 2 of the bottom panel in part D are separated by an intervening lane to prevent any spill over artifacts and other details are same as parts A and B.

It is known that RPA physically associates with ATRIP and since we propose that hSSB1/2-INTS3 complex substitutes the function of RPA, we evaluated the physical interaction between hSSB1/2-INTS3 and ATRIP. Since the antibodies against endogenous proteins immunoprecipitated poorly, we expressed the proteins using affinity tags and assayed the physical interactions. It has been previously shown that the C-terminus of ATRIP (aa 508–776) binds to the N- terminus of ATR (aa 1–388) and this interaction is essential not only for ATR recruitment but also for ATRIP localization to the sites of DNA damage (16). Since the expression of full-length ATR was poor, we tested the interaction of hSSB1-INTS3 with this domain of ATR which is known to be vital for binding to ATRIP. We co-expressed HA-ATR or HA-ATRIP with Myc-INTS3 in 293T cells and observed that HA-ATR as well as HA-ATRIP co-immunoprecipitated Myc-INTS3 indicating *in vivo* interaction between ATR/ATRIP and INTS3 (Figure 5A). We also observed that HA-ATR and HA-ATRIP co-immunoprecipitated Myc-hSSB1 (Figure 5B). To validate the interactions, we carried out reverse immunoprecipitation with anti-Myc antibody: we co-expressed Myc-INTS3 along with HA-ATR or HA-ATRIP and observed that Myc-INTS3 co-immunoprecipitated HA-ATR and HA-ATRIP, authenticating their physical association (Figure 5C and D).

### HSSB1/2-INTS3 recruits ATR-ATRIP complex to single-stranded DNA

**Figure 6.**
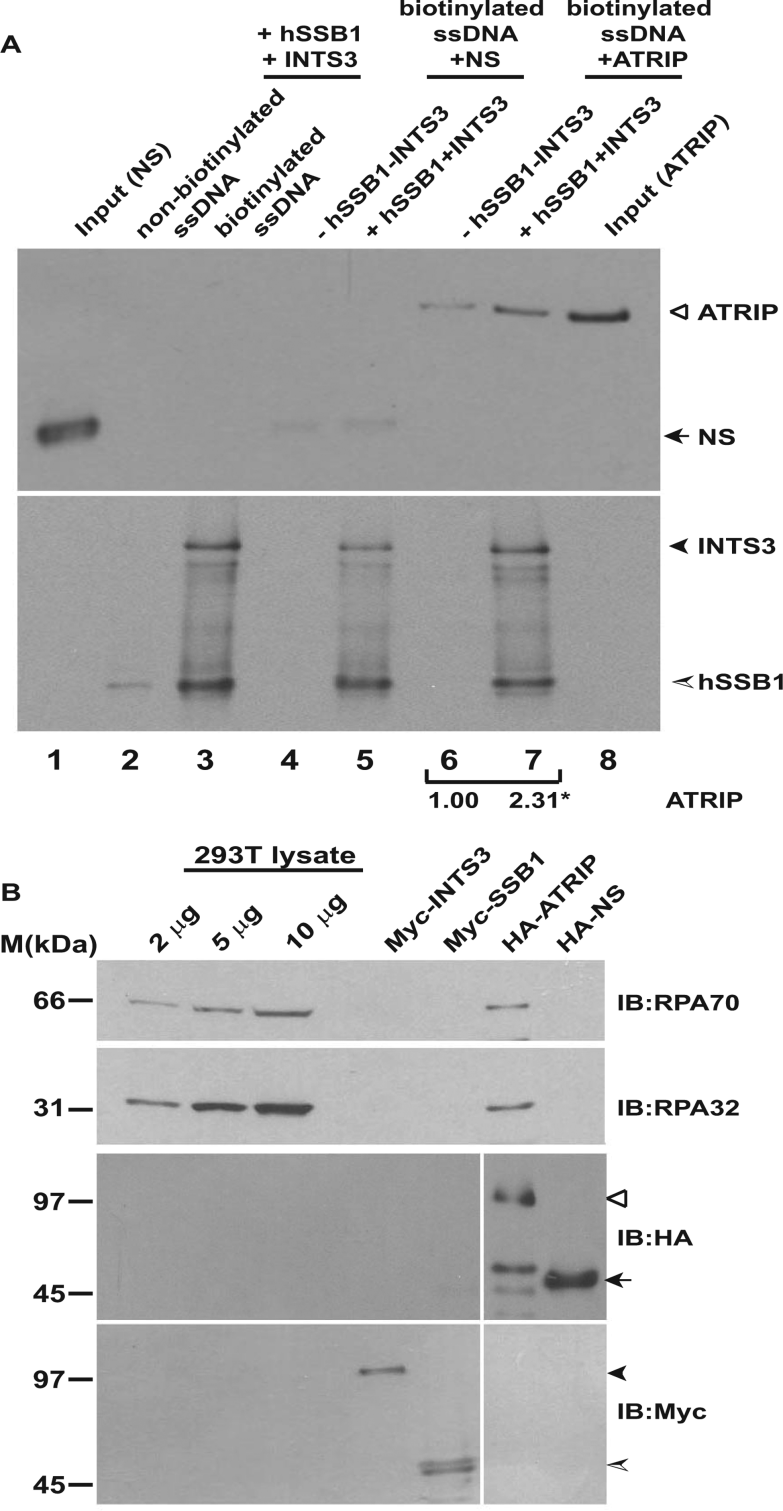
The single-strand binding protein complex, hSSB1/2-INTS3 recruits ATRIP to single-stranded DNA. (**A**) Myc-hSSB1, Myc-INTS3, HA-ATRIP and HA-tagged non-specific control protein (HA-NS) were individually expressed in 293T cells, purified by immunoprecipitation with anti-Myc or anti-HA antibodies and were eluted with Myc or HA peptides. For the single-stranded DNA-binding assay, streptavidin-agarose was incubated with non-biotinylated (lane 2) or biotinylated (lane 3) single-stranded DNA followed by incubation with Myc-hSSB1 and Myc-INTS3. Next, HA-ATRIP or HA-NS purified from 293T cells were incubated with streptavidin-agarose bound biotinylated ssDNA either in the absence (lanes 4 and 6) or presence (lanes 5 and 7) of bound Myc-hSSB1 and Myc-INTS3. After washing, the bound proteins were identified by immunoblotting with anti-HA (top panel) and anti-Myc (bottom panel) antibodies. 10% of NS and ATRIP utilized for binding to streptavidin-agarose has been shown in lanes 1 and 8 respectively and specific proteins have been marked by arrowheads. The control protein (NS) did not bind to hSSB1-INTS3 complex, ruling out non-specific association. The numbers indicate relative binding of HA-ATRIP to ssDNA in the absence or presence of Myc-hSSB1 and Myc-INTS3. *P-value was calculated using two-tailed *t*-test which displays that the ATRIP binding observed in the absence or presence of hSSB1-INTS3 complex is significantly different (*P-value = 0.043). (**B**) RPA complex is absent in Myc-hSSB1 and Myc-INTS3 immunoprecipitates. Myc-hSSB1, Myc-INTS3, HA-ATRIP and HA-NS proteins expressed in 293T cells and purified by elution with Myc or HA peptides following immunoprecipitation were immunoblotted with anti-RPA70 (top panel) and anti-RPA32 (second panel) antibodies for detecting endogenous RPA70 and RPA32. As reported earlier, RPA complex physically associates with ATRIP but is absent from hSSB1-INTS3 complex. Note the high sensitivity of detection of endogenous RPA70 and RPA32 in 293T cell lysate. HA-ATRIP (hollow arrowhead), HA-NS (black arrow) Myc-INTS3 (black arrowhead) and Myc-hSSB1 (shaded arrowhead) have been marked.

We assayed if hSSB1/2-INTS3 complex can promote the binding of ATRIP to ssDNA in an *in vitro* assay, which permits for controlled manipulation of proteins and is impervious to the toxicity that may occur due to *in vivo* depletions. Previous studies have addressed the effect of RPA on ATRIP binding to ssDNA: while one study reported that ATRIP efficiently bound to ssDNA only in the presence of RPA, another study reported that there was no significant difference in ATRIP binding to both naked and RPA-coated ssDNA and it has been proposed that a distinct ATRIP-ssDNA binding mode exists, that does not require RPA (11,35,36). Myc-hSSB1, Myc-INTS3, HA-ATRIP and a non-specific control protein (HA-NS) were purified from 293T cells. Streptavidin-agarose was incubated with biotinylated or non-biotinylated single-stranded DNA followed by incubation with Myc-hSSB1 and Myc-INTS3. Purified hSSB1 and INTS3 bound to streptavidin-agarose if coated with biotinylated ssDNA indicating that hSSB1-INTS3 complex specifically binds to ssDNA (Figure 6A, lane 3, bottom panel). Next, we incubated HA-ATRIP with biotinylated ssDNA either in the absence or presence of the bound hSSB1-INTS3 complex. As reported by other groups, we also observed low affinity ATRIP binding to ssDNA *in vitro* (lane 6, top panel) (35,36). Binding of ATRIP to ssDNA was enhanced by 2.6 fold if it was coated with hSSB1-INTS3 complex, demonstrating that ATRIP can be recruited to ssDNA by hSSB1-INTS3 complex (compare lanes 6 and 7, top panel). To rule out the possibility that enhanced ATRIP binding to hSSB1-INTS3 coated ssDNA is due to contaminating RPA, the purified proteins were immunoblotted for detecting endogenous RPA70 and RPA32 (Figure 6B). As reported earlier, RPA complex physically associates with ATRIP but is absent in hSSB1 and INTS3 eluates, ruling out the possibility that enhanced ATRIP binding is due to contaminating RPA (35,36).

### INTS3 mediates the recruitment of ATRIP to the sites of genomic stress

**Figure 7.**
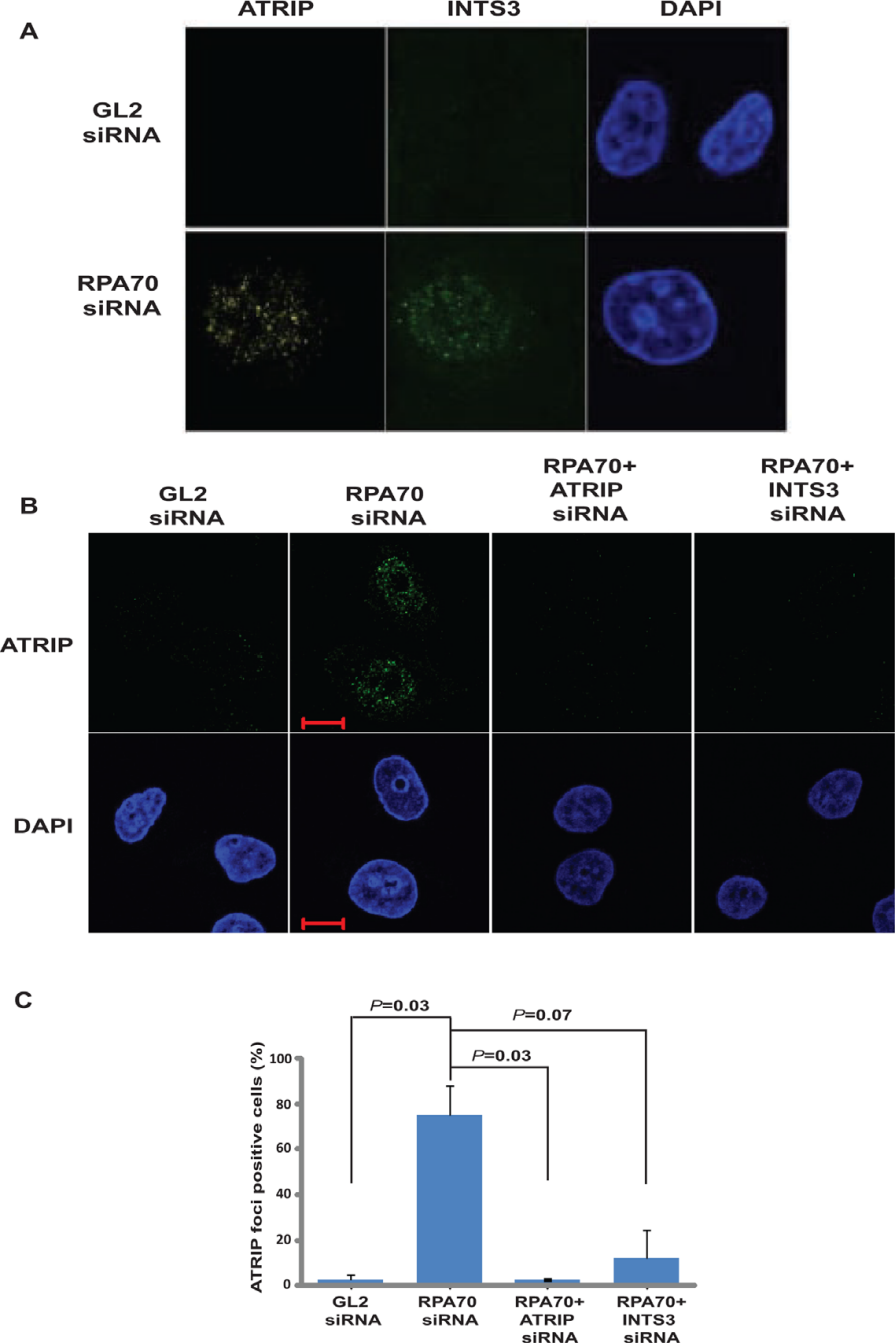
INTS3 is required for formation of ATRIP foci after RPA70 depletion. (**A**) HeLa cells transfected with *GL2* or *RPA70* siRNA were visualized for ATRIP (left panel) and INTS3 (middle-left panel) foci by co-immunofluorescence using rabbit anti-ATRIP and goat anti-INTS3 antibodies in combination with anti-rabbit Alexa Fluor 594 and anti-goat Alexa Fluor 488 antibodies respectively. (**B**) HeLa cells transfected on three consecutive days with *GL2* or *RPA70* siRNA in combination with *ATRIP* or *INTS3* siRNA as indicated were visualized for ATRIP foci by immunofluorescence with rabbit anti-ATRIP antibody. Bottom panel displays the DAPI staining of each sample. Note that ATRIP foci formed after RPA70 depletion (second panel) are absent after INTS3 depletion (fourth panel). Co-depletion of ATRIP along with RPA70 (third panel) confirms that the observed immunofluorescence signal is from ATRIP. The scale bar is 10 μm. (**C**) Quantification of ATRIP foci observed in the experiment described in part B. Cells were scored for ATRIP foci and expressed as a percentage of total cells from each transfected sample. Data are represented as the mean ± SE. *P-*values of *RPA70* siRNA transfected samples versus other groups have been indicated.

It is known that ATRIP localizes to the sites of DNA damage to initiate the checkpoint pathway (16). After RPA70 depletion, we checked the localization of endogenous ATRIP and observed that it forms punctate foci, typical of recruitment to the sites of DNA damage (Figure 7A). It has been previously reported that hSSB1/2-INTS3 displays only partial co-localization with other repair proteins such as MRN or Rad51 and further studies are required to understand the nature of ATRIP and INTS3 foci (23,27,29). To assess the role of alternate SSB in ATRIP recruitment, we co-depleted INTS3 along with RPA70: we observed that ATRIP foci formed after RPA70 depletion were dissipated in the absence of INTS3 (Figure 7B and C and Supplementary Figure S4). Therefore, we infer that hSSB1/2-INTS3 complex recruits ATRIP to the sites of genomic stress.

### Inactivation of RPA along with hSSB1/2-INTS3 complex incapacitates the ability of cells to phosphorylate Chk1

**Figure 8.**
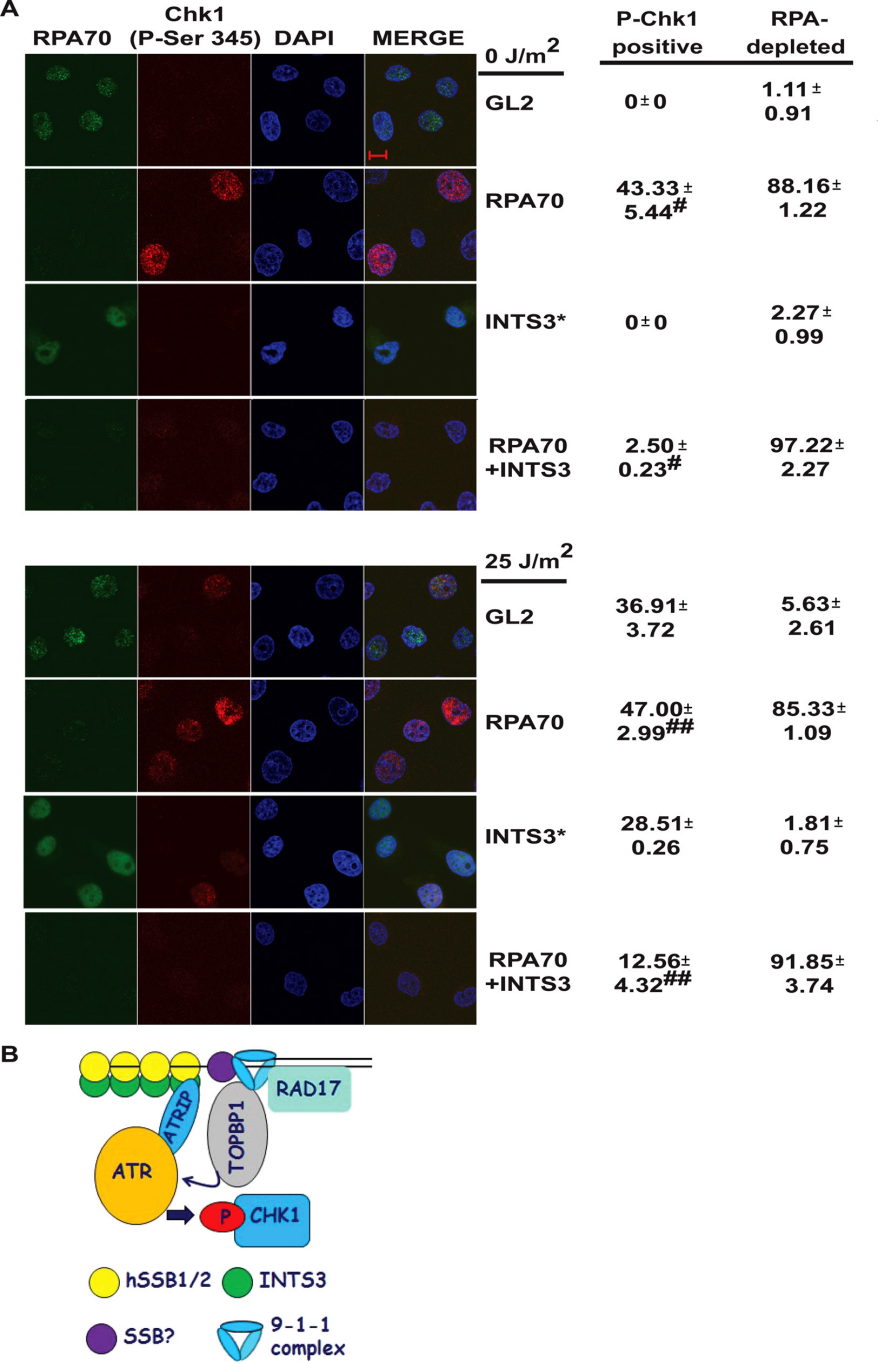
Inactivation of hSSB1/2-INTS3 complex along with RPA debilitates the ability of cells to phosphorylate Chk1. (**A**) HeLa cells were transfected on three consecutive days with *GL2* or *RPA70* siRNA in combination with *INTS3* siRNA, as indicated. 24 h after the last transfection, the cells were either left non-irradiated (top four panels) or UV-irradiated (bottom four panels) as indicated, followed by co-immunofluorescence with mouse anti-RPA70 and rabbit anti-phospho-Chk1 (Ser345) antibodies. The scale bar is 10 μm. Cells were scored for RPA70 and P-Chk1 signals and the percentage of total cells from each group displaying RPA70 depletion and phosphorylated-Chk1 have been indicated. All data represent the mean ± SE of two independent experiments. * indicates that the siRNA depletion was carried out in a separate experiment wherein 38.45% of UV-irradiated *GL2* cells displayed phosphorylated-Chk1. ^#^*P-*values were calculated using two-tailed *t*-test which displays that the Chk1 phosphorylation observed in the non-UV-irradiated *RPA70* siRNA transfected sample is significantly different from *RPA70+INTS3* siRNA transfected sample (^#^*P-*value = 0.026). ^##^ The *P-*value of UV-irradiated *RPA70* siRNA transfected sample versus *RPA70+INTS3* siRNA transfected sample is 0.033. (**B**) A model for ATR-ATRIP recruitment by hSSB1/2-INTS3 complex. Single-stranded DNA (ssDNA) generated at the sites of genomic stress is coated by hSSB1/2-INTS3 complex in the absence of RPA. The N-terminus of INTS3 associates with the oligonucleotide/oligosaccharide-binding fold of hSSB1/2, which binds to the ssDNA (23,25–27). ATR-ATRIP complex is then recruited to the hSSB1/2-INTS3 bound ssDNA. Rad9-Hus1-Rad1 clamp (9–1–1 complex) is recruited to ssDNA independently of ATRIP but it is not clear which single-stranded DNA-binding protein (SSB?) associates with it in the absence of RPA. TopBP1 binds to Rad17 loaded Rad9 to come in close proximity to activate ATR, which phosphorylates Chk1. It is likely that many yet unidentified factors participate in RPA-independent ATR activation.

HSSB1/2-INTS3 serves as the single-strand binding protein complex in RPA-depleted cells, and we wanted to evaluate the cell's ability to phosphorylate Chk1 in response to an exogenous genomic stress, when both complexes were inactivated. First, we depleted RPA70 and INTS3 and assayed for Chk1 phosphorylation in individual cells by immunofluorescence. None of the control siRNA transfected cells displayed phosphorylated-Chk1 signal, while around 43% of *RPA70* siRNA transfected cells were positive for Chk1 phosphorylation (Figure 8A, panel 2). When INTS3 was depleted along with RPA70 only 2% of the cells were positive for phosphorylated-Chk1 (panel 4). When control siRNA transfected cells were exposed to exogenous genomic stress caused by UV, approximately 37% of cells were positive for Chk1 phosphorylation (panel 5). It was reduced to approximately 28% in the absence of INTS3, indicating that UV radiation-induced Chk1 phosphorylation was partially initiated by resection of the DSBs (panels 5 and 7). On UV-irradiating RPA70-depleted cells, 47% cells were positive for Chk1 phosphorylation that was reduced to 12% when INTS3 was co-depleted along with RPA70 (panels 6 and 8). It should be noted that RPA70 depletion caused an increase in an S-phase from around 22% to 48%, resulting in a higher fraction of cells with Chk1 phosphorylation (Figure 1C, 1D and Supplementary Figure S1D). When *RPA70* siRNA transfected cells were pulsed with BrdU before exposure to UV radiation most of the cells that underwent Chk1 phosphorylation also incorporated BrdU, which independently demonstrates that Chk1 phosphorylation in RPA70-depleted UV-irradiated cells occurs during the S-phase (Supplementary Figure S5). Therefore, co-depletion of RPA70 and INTS3 incapacitates the ability of cells to phosphorylate Chk1 in response to exogenous genomic stress.

### HSSB1/2-INTS3 complex is required for Chk1 phosphorylation in RPA proficient cells

**Figure 9.**
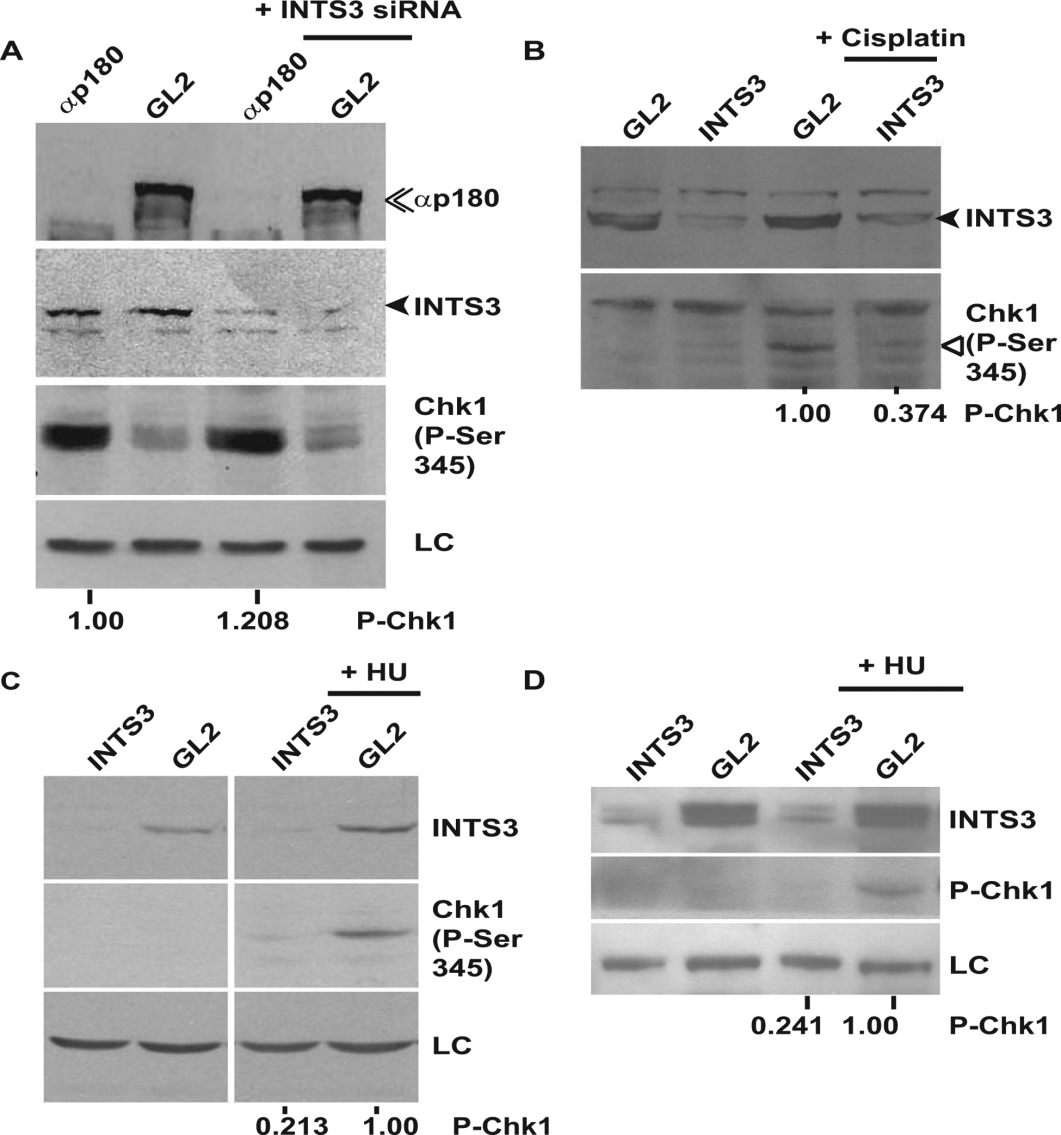
INTS3 is essential for hydroxyurea (HU)- and cisplatin-induced Chk1 phosphorylation in RPA proficient cells. (**A**) HeLa cells were transfected on three consecutive days with *GL2* or siRNA targeting the catalytic subunit of DNA polymerase alpha (αp180) in combination with *INTS3* siRNA as indicated and the levels of αp180 (double arrowhead), phosphorylated-Chk1 and INTS3 (black arrowhead) were assayed. LC indicates the protein loading control. The numbers indicate phosphorylated-Chk1 levels following αp180 depletion alone or in combination with INTS3 after normalization with the protein loading control. (**B**) INTS3 is essential for cisplatin-induced Chk1 phosphorylation. HeLa cells transfected on three consecutive days with *GL2* or *INTS3* siRNA were exposed to 0.2 μM cisplatin and harvested after 8 h. The levels of INTS3 (black arrowhead) and phosphorylated-Chk1 (hollow arrowhead) were assayed and the numbers indicate phosphorylated-Chk1 levels after normalization with the protein loading control. HeLa (**C**) and human foreskin fibroblast (**D**) cells transfected on three consecutive days with *GL2* or *INTS3* siRNA were treated with 0.2 mM HU and harvested after 4 h. The numbers indicate phosphorylated-Chk1 levels in HU treated cells transfected with *GL2* or *INTS3* siRNA after normalization with the protein loading control.

We have shown that hSSB1/2-INTS3 complex is required for Chk1 phosphorylation after RPA depletion and finally we wanted to evaluate its role in checkpoint activation induced by other genotoxic agents. We depleted the catalytic subunit of DNA Polymerase alpha (αp180), which also leads to replication fork stalling, resulting in Chk1 phosphorylation (Figure 9A). We observed that co-depletion of INTS3 along with αp180 did not suppress phosphorylation of Chk1, indicating that the replication stalling-induced Chk1 phosphorylation is not dependent on hSSB1-INTS3 complex in RPA proficient cells. Treatment with cisplatin leads to intrastrand and interstrand DNA cross-links, resulting in DNA breaks and we observed that INTS3 depletion suppressed the cisplatin-induced Chk1 phosphorylation in HeLa cells (Figure 9B). Hydroxyurea (HU) treatment at high concentrations leads to replication fork collapse resulting in DNA breaks and we observed that INTS3 depletion suppressed HU-induced Chk1 phosphorylation in both HeLa cells and human foreskin fibroblasts, demonstrating its essential role in Chk1 activation in RPA proficient cells, as has also been reported recently (Figure 9C and D) (30). Though future investigations would discern the recruitment of hSSB1/2-INTS3 complex to the sites of DNA damage in response to different genotoxic agents, it seems that Chk1 phosphorylation resulting from DNA breaks is inhibited by the depletion of hSSB1/2-INTS3 due to its role in DNA strand break repair pathway.

## DISCUSSION

We hypothesize that ATR-ATRIP can bind to DNA by two mechanisms: the first is RPA-dependent and the second requires the hSSB1/2-INTS3 complex (model in Figure 8B). In case the RPA-dependent complex is non-functional, the alternate complex may serve to activate the checkpoint during genomic stress. To understand how hSSB1/2-INTS3 complex would recruit ATR-ATRIP complex, cues can be taken from our understanding of RPA binding to ATRIP. The N-terminus OB fold of RPA70 binds to ATRIP, the centrally located OB folds bind to ssDNA while the C-terminus OB fold interacts with RPA32, which assembles the complex by binding to RPA14 (37–39). ATRIP and Rad9 associate with adjacent ssDNA-bound RPA complexes bringing TopBP1 in close proximity to activate ATR (15). It has been shown that the N-terminus of INTS3 directly binds to the OB fold of hSSB1/2 and since INTS3 does not have any recognizable OB fold, the interaction with ssDNA is likely to be mediated by hSSB1/2 (27). We believe that after the hSSB1/2-INTS3 complex has been assembled, it physically associates with ATRIP. It is important to note that the other factors required for this novel mode of ATR activation are yet to be identified. Since RPA70 is also required for Rad9 recruitment, further studies are needed to determine if hSSB1/2-INTS3 complex can recruit Rad9 (27,36). Therefore, the change from the canonical activation of ATR is replacement of RPA by hSSB1/2-INTS3 complex while other factors remain the same. It has been observed that the dissociation constant of purified hSSB1 for ssDNA *in vitro* is close to 10^−6^ M, indicating that affinity of hSSB1 for ssDNA is much lower than RPA (23). Since hSSB1/2 is recruited to the damage foci only in the presence of INTS3, it is likely that the *in vivo* association between INTS3 and hSSB1/2 enhances the ssDNA binding (25,26). Moreover, it has been observed that the binding of hSSB1 increases with the length of ssDNA, we believe that longer segments of ssDNA generated during stress would be coated by the hSSB1/2-INTS3 complex. Cimprich and coworkers have also observed that a distinct ATR-ATRIP complex could bind to DNA with low affinity in the absence of RPA (36).

It has been previously reported that loss of MRN complex does not alter the stress-induced Chk1 phosphorylation though other studies have clearly shown that MRN interacts and co-localizes with RPA at the sites of stalled replication forks and cells deficient in MRN activity display defective intra-S-phase checkpoint (40–44). It has been demonstrated that cells deficient in Nbs1 activity have defective Chk1 phosphorylation in response to hydroxyurea and UV radiation and these cells fail to retain ATR at the sites of DNA damage (45). Using *Xenopus* nuclear extracts, recent work has comprehensively addressed this issue by demonstrating that MRN complex was enriched on ATR-activating structures and its depletion markedly decreased the binding of TopBP1 with the checkpoint-activating structure and abrogated the phosphorylation of Chk1 (46,47). Another recent study reported that in Rad50-deficient cells ATRIP and RPA do not co-localize after HU treatment resulting in poor recruitment of ATR to the sites of replication stress thus failing to induce Chk1 phosphorylation (48). However, in our study, Chk1 phosphorylation was not abrogated after Mre11 or Nbs1 depletion. Though future investigations would be required to ascertain the reasons for this difference, it is possible that RNAi does not completely annul the MRN activity. It is also conceivable that the alternate mode of ATR activation utilizes distinct pathways for recruiting adaptor checkpoint proteins, though such pathways are yet to be identified.

Though we show that human SSB1/2-INTS3 complex can perform the function of recruiting the ATRIP-ATR complex to ssDNA, its requirement may vary across eukaryotes: It has been reported that absence of RPA decreases the binding of budding yeast and *Xenopus* orthologs of ATRIP or ATR to ssDNA (11,49,50). SSB1 is not conserved in budding yeast and therefore, it is possible that higher eukaryotes have evolved multiple SSBs for responding to stress. INTS3 enhances the transcription of SSB1, which leads to an increase in SSB1 protein and its localization to the sites of damage (25). Such a regulatory loop is unlikely to manifest in a short *Xenopus* cell-free assay and therefore, it is possible that in such a system RPA is essential for the association of ATR. We believe that the different requirements of RPA reflect varying presence of alternate factors that can substitute the canonical RPA-ATRIP-ATR interactions.

Though RPA is an essential protein complex, multiple instances of its partial loss have been reported, wherein the hSSB1/2-INTS3-ATR pathway may be functional. To address the effect of partial loss of RPA function, a mouse cell line with a hypomorphic mutation in RPA70 (L230P) was generated which causes sensitivity to DNA-damaging agents without affecting DNA replication (51,52). Offsprings heterozygous for this RPA70 mutation were viable but homozygous mutant mice were embryonically lethal. RPA70 heterozygous mutant mice displayed widespread chromosomal rearrangements and developed lymphomas retaining both the mutant and the wild-type allele suggesting that the mutated allele had a dominant effect. The study demonstrated that though the complete loss of RPA70 function is not tolerated by cells, heterozygous RPA70 mutants are viable and we believe that in such physiological states where the checkpoint function of RPA70 is not optimal, the hSSB1/2-INTS3-ATR pathway could be functional.

Haploinsufficiency of RPA70 has also been reported clinically: deletions within 17p13.3 chromosomal region, which harbors the RPA70 gene is associated with clinical features including developmental delay and autism spectrum disorder known as Miller-Dieker syndrome (53,54). Cell lines from these patients display reduced levels of RPA70 protein which is consistent with the deletion mapping (55). There is strong evidence that haploinsufficiency of components of the ATR-dependent signaling pathway, including ATR and RPA70, results in the impaired ability of these checkpoint proteins in responding to genotoxic stress. It is unclear if 17p13.3 deletion predisposes cells to cancer though isolated cases of malignancy has been reported in patients of Miller-Dieker syndrome and it is therefore possible that loss of RPA checkpoint function is compensated by hSSB1/2-INTS3 pathway (56). Therefore, the role of hSSB1/2-INTS3 complex in ATR activation in cells lines displaying reduced levels of RPA70 activity should be investigated: it should be ascertained if inactivation of hSSB1/2-INTS3 in RPA mutant cell lines leads to further loss of checkpoint activity and oncogenesis. It should be pointed out that deletions of hSSB2 gene have been reported in medulloblastoma and renal cancers, though their clinical significance is yet to be established.

Finally, we have also shown data which demonstrate that INTS3 depletion suppresses HU- and cisplatin- induced Chk1 phosphorylation in both primary and transformed cells illustrating that the hSSB1/2-INTS3 complex has a physiological role in responding to genomic stress caused by different genotoxic agents (Figure 9). It is unlikely that loss of RPA would be tolerated by eukaryotic cells but by depleting it experimentally, we have discovered an alternate mechanism that activates ATR signaling. A recent study has shown that hSSB1 (hSSB1^−/−^) deleted mouse displays chromosomal instability without any defect in the ATM arm of checkpoint activation (57). Since the genomic fragmentation observed in hSSB1^−/−^ mouse was very similar to what has been reported for ATR deficiency, we predict that the loss of hSSB1-mediated ATR activation may be the reason for the observed genomic instability (57,58). In summation, we demonstrate that an alternate single-stranded DNA-binding protein complex can recruit the principal checkpoint transducer, the ATR-ATRIP complex to initiate ATR signaling.

## SUPPLEMENTARY DATA

Supplementary Data are available at NAR Online.

SUPPLEMENTARY DATA
