# Supplementary material for: RPA70 depletion induces hSSB1/2-INTS3 complex to initiate ATR signaling
Source: Nucleic Acids Res. 2015 Apr 27;43(10):4962–74. doi: 10.1093/nar/gkv369 (PMC4446429; doi:10.1093/nar/gkv369)
Supplement: SUPPLEMENTARY DATA [file supp_43_10_4962__index.html]

RPA70 depletion induces hSSB1/2-INTS3 complex to initiate ATR signaling — RPA70 depletion induces hSSB1/2-INTS3 complex to initiate ATR signaling — SUPPLEMENTARY DATA 

# RPA70 depletion induces hSSB1/2-INTS3 complex to initiate ATR signaling

## SUPPLEMENTARY DATA

**Files in this Data Supplement:**

- SUPPLEMENTARY DATA
