# Supplementary material for: RPA70 depletion induces hSSB1/2-INTS3 complex to initiate ATR signaling
Source: Nucleic Acids Res. 2015 Apr 27;43(10):4962–74. doi: 10.1093/nar/gkv369 (PMC4446429; doi:10.1093/nar/gkv369)

SUPPLEMENTARY INFORMATION

Supplementary Figure S1. RPA70 depletion-induced Chk1 phosphorylation. (A) To confirm the phosphorylation of Chk1 at Ser345 and Ser317, HeLa cells transfected with control *GL2* or *RPA70* siRNA were immunoblotted with different anti-phospho-Chk1 (Ser345 and Ser317) antibodies than those used in Figure 1A. Black and hollow arrowheads point to phosphorylated-Ser345 and phosphorylated-Ser317 Chk1 bands respectively. LC refers to the protein loading control. (B) RPA70 depletion causes phosphorylation of Chk1 in cell lines from different lineages. HCT116, 293T and U2OS cells were transfected on three consecutive days with control *GL2* or *RPA70* siRNA and the levels of RPA70 and phosphorylated-Chk1 (Ser345) were assayed. (C) Overexpression of siRNA resistant RPA70 suppresses the *RPA70* siRNA depletion-induced phosphorylation of Chk1. 293T cells were transfected on three consecutive days with control *GL2* or *RPA70* siRNA (which targets 291-310 nt of 3' UTR region of *RPA70* mRNA) in combination with plasmids expressing either HA-tagged coding region of RPA70 (HA-RPA70) or a non-specific protein (HA-NS; marked by arrow). Apart from full length protein (marked by single arrowhead on the left side of the panel) exogenous expression of RPA70 also generates shorter forms of the protein (double-arrowhead). Note that all forms of HA-RPA70 are recognized by the endogenous RPA70 antibody (black arrowhead) and since the mobility of the shorter form of HA-RPA70 is similar to the endogenous RPA70, it masks the RPA70 depletion. The depletion of RPA70 is less efficient, probably due to the co-transfecting DNA plasmid. The numbers indicate phosphorylated-Chk1 levels following RPA70 depletion alone or in co-expression with HA-RPA70 after normalization with the protein loading control. Note that Chk1 phosphorylation (hollow arrowhead) is suppressed by siRNA resistant HA-RPA70.(D) Cell cycle distribution after RPA depletion and mevastatin block. The top two histograms displays asynchronous HeLa cells (Asn) transfected on three consecutive days with *GL2* or *RPA70* siRNA and stained with propidium iodide for flow cytometry analysis. Independently, cells blocked in G1-phase with mevastatin were transfected with *RPA70* siRNA for three consecutive days and the cells were harvested in the presence of mevastatin (Mev-0 h) (bottom left histogram). A subset of *RPA70* siRNA transfected and mevastatin blocked cells were released from the cell-cycle block by removing mevastatin and harvested after 12 h (Mev-12 h). Flow cytometry of propidium iodide stained DNA from HeLa cells demonstrates that after removal of mevastatin, the blocked cells slowly move into the S phase. Inset displays distribution of cells in G1, S and G2 phases.

Supplementary Figure S2. Requirement of hSSB1/2-INTS3 complex in RPA-independent Chk1 phosphorylation. (A) As explained in Figure 2, HeLa cells transfected with control *GL2* or *RPA70* siRNA were visualized for INTS3 and hSSB1 foci. INTS3 and hSSB1 foci observed in an independent experiment than that described in Figure 2 A and B have been shown. (B) As reported previously, INTS3 depletion inhibits gamma radiation-induced checkpoint response (27). HeLa cells transfected on three consecutive days with *GL2* or *INTS3* siRNA were exposed to 10 Gy gamma radiation and the levels of INTS3 and phosphorylated-Chk1 were assayed. LC indicates the protein loading control. The numbers indicate phosphorylated-Chk1 levels (P-Chk1) relative to gamma-irradiated *GL2* siRNA transfected cells after normalization with the protein loading control.

Supplementary Figure S3. Independent siRNA duplexes confirm the requirement of the ATR-ATRIP complex in RPA-independent Chk1 phosphorylation. (A-D) HeLa cells were transfected on three consecutive days with *GL2* or *RPA70* siRNA in combination with different siRNA duplexes (*ATRIP(2)*, *ATR(2)*, *TOPBP1(2)* or *RAD17(2)*) than used in Figure 4. LC refers to the protein loading control. The numbers in parts A to D indicate phosphorylated-Chk1 levels following RPA70 depletion alone or in combination with other proteins after normalization with the protein loading control. (E) The decrease

of *RAD17* mRNA was confirmed by reverse-transcriptase PCR and the numbers indicate the mRNA level following specific siRNA depletion relative to control *GL2* transfected cells. β-2 microglobulin (BMG) serves as the internal RNA loading control.

Supplementary Figure S4. Independent siRNA duplexes confirm the requirement of INTS3 in the formation of ATRIP foci after RPA70 depletion. (A) HeLa cells were transfected on three consecutive days with *GL2* or *RPA70* siRNA in combination with a different *INTS3* siRNA duplex (*INTS3(2)*) than used in Figure 7B and visualized for ATRIP foci by immunofluorescence with rabbit anti-ATRIP antibody.

Supplementary Figure S5. Chk1 phosphorylation in RPA-depleted UV-irradiated cells occurs during DNA replication. (A) Schematic representation of the experiment reported in parts B and C. HeLa cells transfected on three consecutive days with *RPA70* siRNA were pulsed with 100 µM BrdU for 30 min, washed and exposed to 25 J/m^2 UV followed by fixation after 120 min. (B) Fixed HeLa cells were visualized for BrdU incorporation and Chk1 phosphorylation by co-immunofluorescence with mouse anti-BrdU and rabbit anti-phospho-Chk1 (Ser345) antibodies in combination with anti-mouse Alexa Fluor 488 and anti-rabbit Alexa Fluor 555 antibodies respectively. The arrangement of immunofluorescence images obtained from a single field has been illustrated in the top-left panel. Bottom right square of each field is a merge of Alexa Fluor 488, Alexa Fluor 555 and DAPI images. Cell marked as ‘1’ is positive for Alexa Fluor 488 (BrdU) and Alexa Fluor 555 (phospho-Chk1) signal indicating that Chk1 phosphorylation coincides with active DNA replication. To demonstrate that there is no bleed-through of fluorescent signals, a subset of cells were either pulsed for BrdU but not UV-irradiated (Cell marked as ‘2’) or not pulsed for BrdU but were UV-irradiated (Cell marked as ‘3’) followed by co-immunofluorescence with mouse anti-BrdU and rabbit anti-phospho-Chk1 (Ser345) antibodies. (C) RPA-depleted UV-irradiated cells were scored for BrdU and phospho-Chk1 signals and expressed as a percentage of total cells. Note that most of the phospho-Chk1 positive cells are also positive for BrdU signal.

Supplemental Figure S1

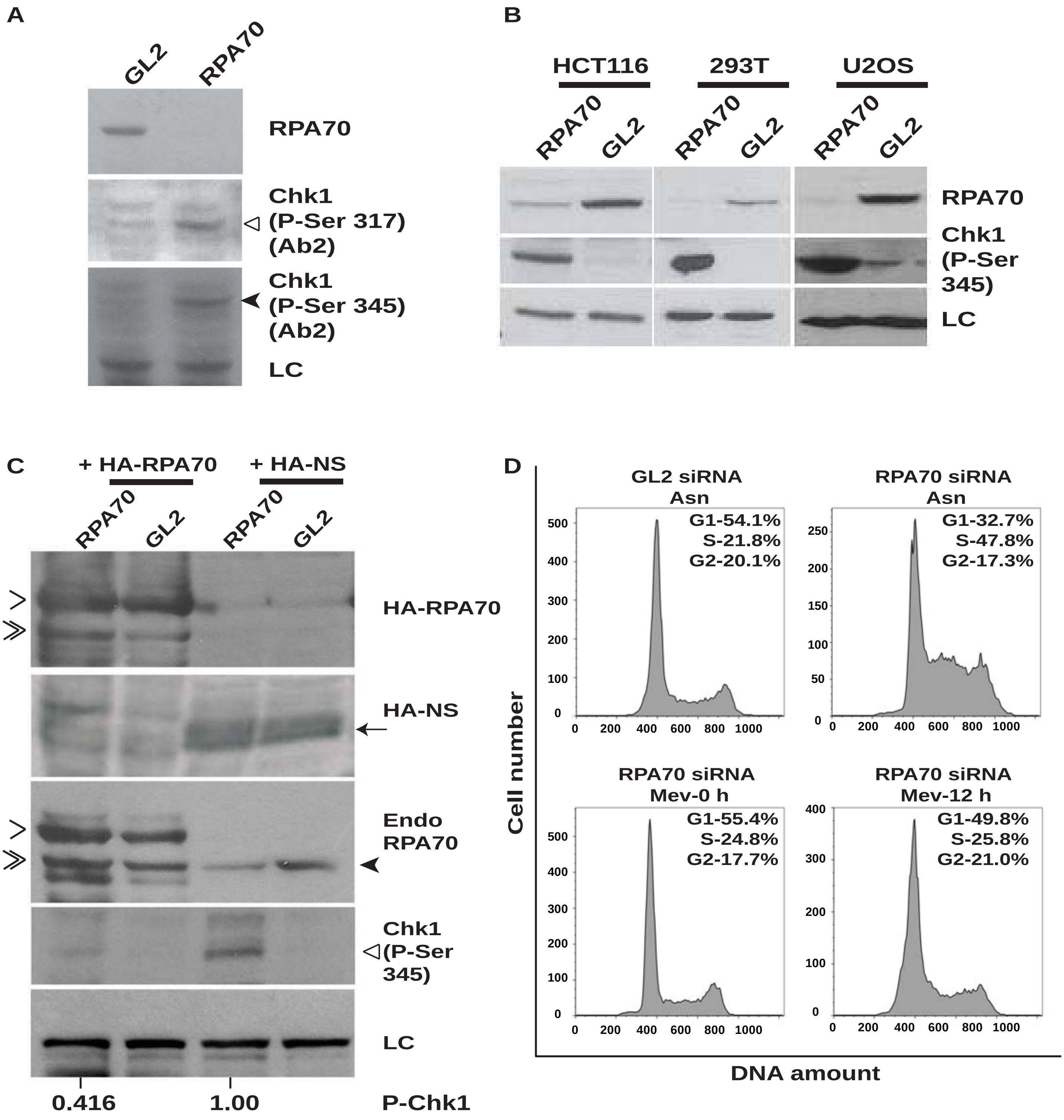

Supplemental Figure S2

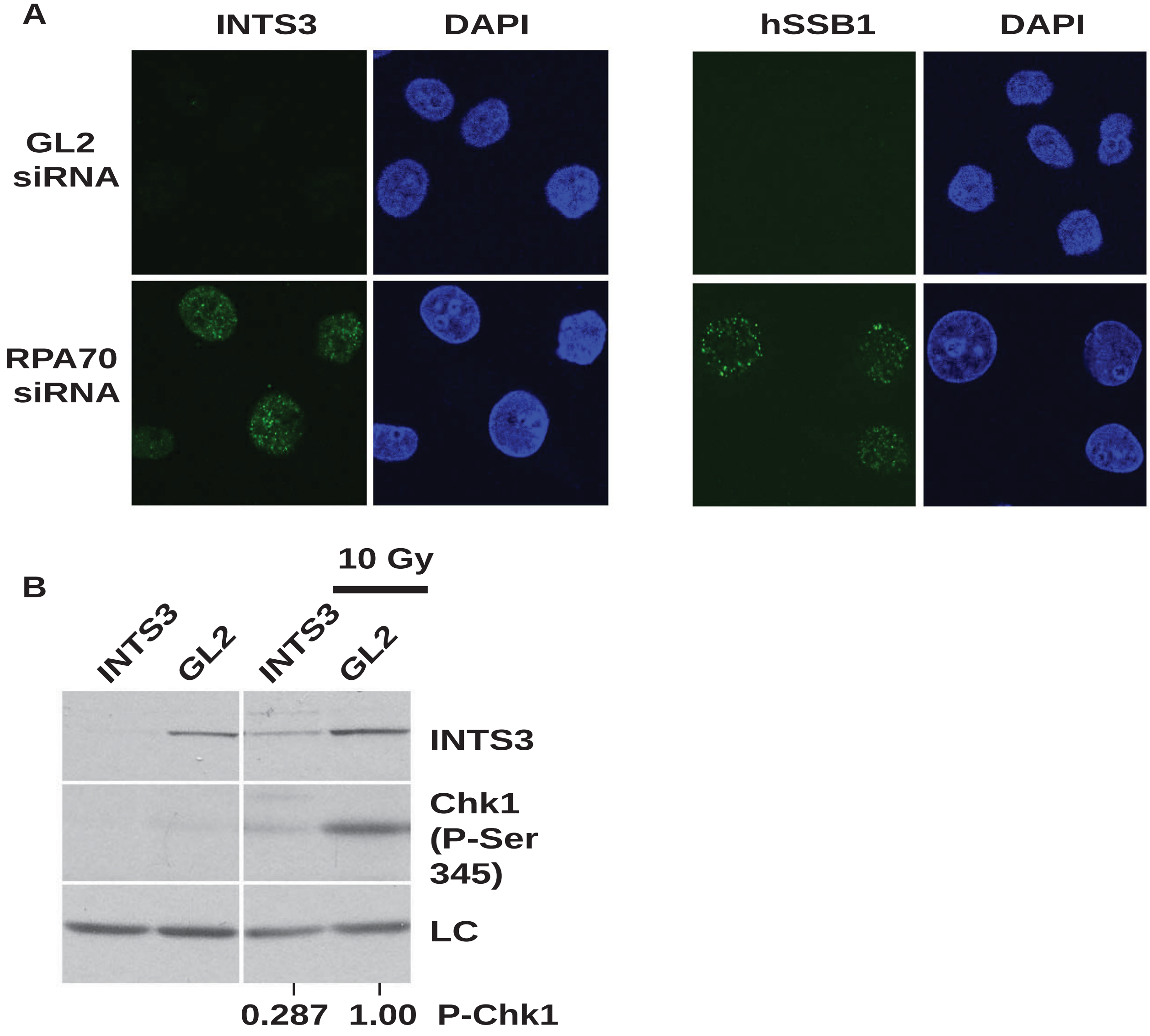

Supplemental Figure S3

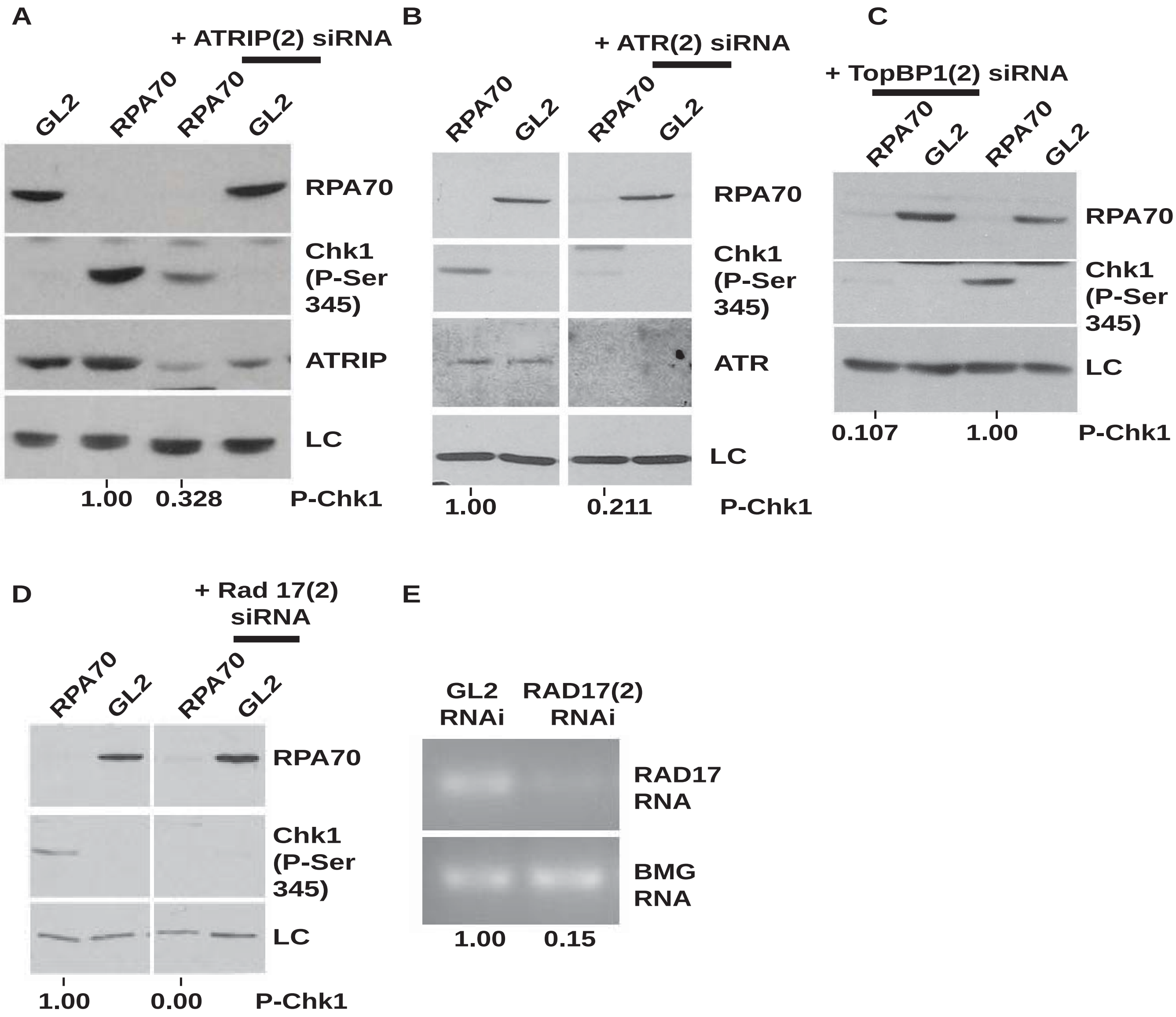

Supplemental Figure S4

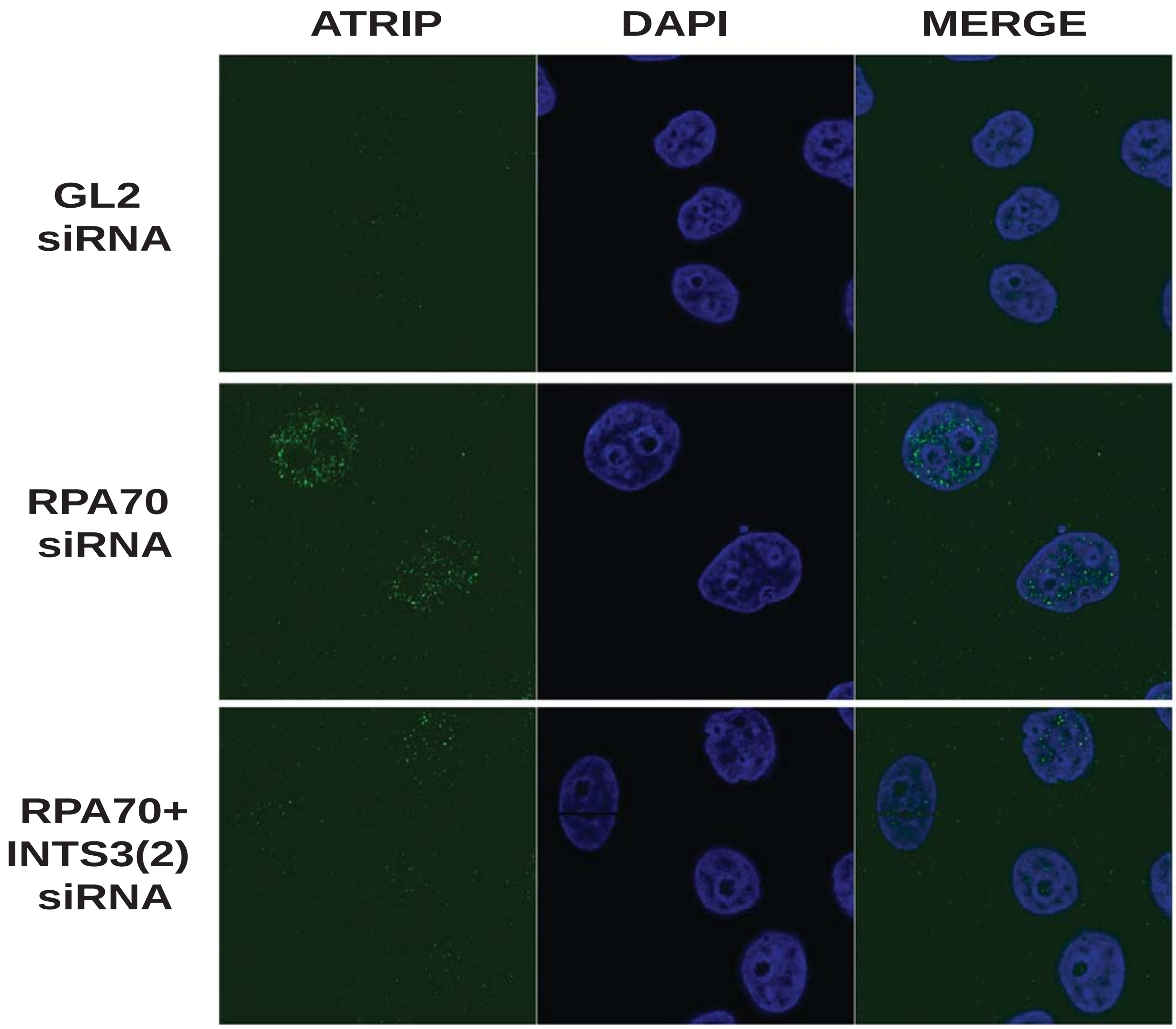

Supplemental Figure S5

A

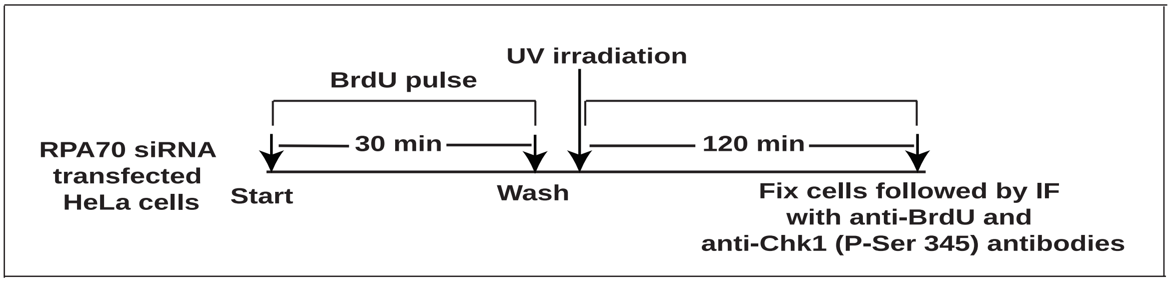

B

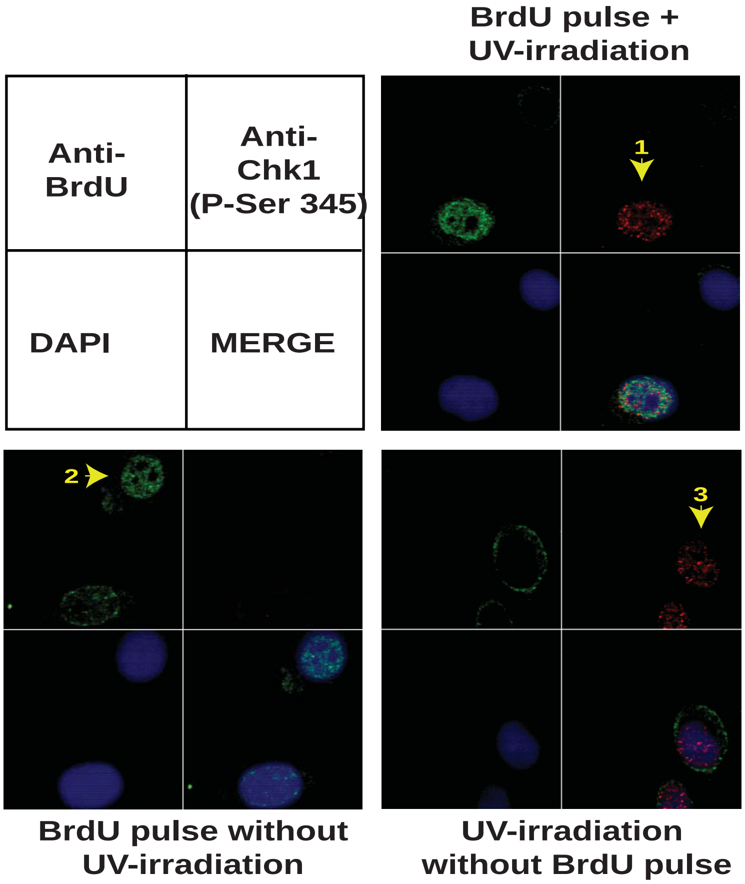

C

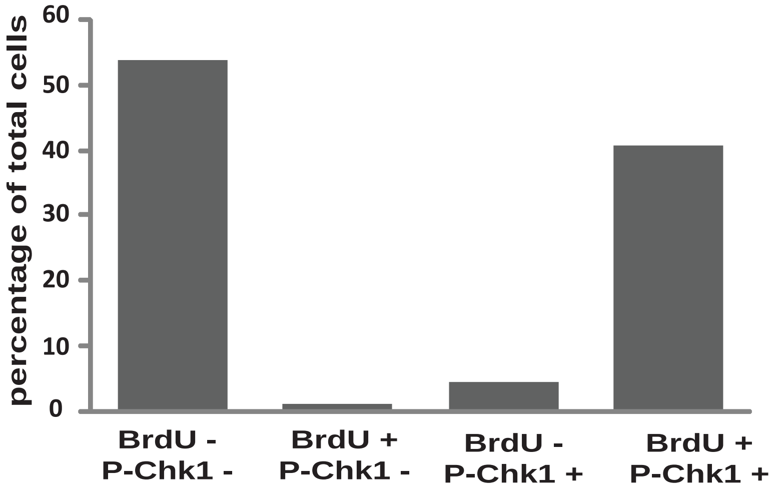

Supplement: SUPPLEMENTARY DATA [file supp_gkv369_nar-00078-x-2015-File011.pdf]
